# Proteome Analysis of Pancreatic Tumors Implicates Extracellular Matrix in Patient Outcome

**DOI:** 10.1158/2767-9764.CRC-21-0100

**Published:** 2022-06-14

**Authors:** Laxmi Silwal-Pandit, Stina M. Stålberg, Henrik J. Johansson, Georgios Mermelekas, Inger Marie B. Lothe, Martina L. Skrede, Astrid Marie Dalsgaard, Daniel J. H. Nebdal, Åslaug Helland, Ole Christian Lingjærde, Knut Jørgen Labori, Bjørn S. Skålhegg, Janne Lehtiö, Elin H. Kure

**Affiliations:** 1 Department of Cancer Genetics, Institute for Cancer Research, Oslo University Hospital, Oslo, Norway.; 2 Department of Natural Sciences and Environmental Health, University of South-Eastern Norway, Bø i Telemark, Norway.; 3 Department of Oncology-Pathology, Karolinska Institutet, Science for Life Laboratory, Solna, Sweden.; 4 Department of Pathology, Oslo University Hospital, Oslo, Norway.; 5 Institute of Clinical Medicine, University of Oslo, Oslo, Norway.; 6 Department of Computer Science, University of Oslo, Oslo, Norway.; 7 Department of Hepato-Pancreato-Biliary Surgery, Oslo University Hospital, Oslo, Norway.; 8 Division of Molecular Nutrition, University of Oslo, Oslo, Norway.

## Abstract

Pancreatic cancer remains a disease with unmet clinical needs and inadequate diagnostic, prognostic, and predictive biomarkers. In-depth characterization of the disease proteome is limited. This study thus aims to define and describe protein networks underlying pancreatic cancer and identify protein centric subtypes with clinical relevance. Mass spectrometry–based proteomics was used to identify and quantify the proteome in tumor tissue, tumor-adjacent tissue, and patient-derived xenografts (PDX)-derived cell lines from patients with pancreatic cancer, and tissues from patients with chronic pancreatitis. We identified, quantified, and characterized 11,634 proteins from 72 pancreatic tissue samples. Network focused analysis of the proteomics data led to identification of a tumor epithelium–specific module and an extracellular matrix (ECM)-associated module that discriminated pancreatic tumor tissue from both tumor adjacent tissue and pancreatitis tissue. On the basis of the ECM module, we defined an ECM-high and an ECM-low subgroup, where the ECM-high subgroup was associated with poor prognosis (median survival months: 15.3 *vs*. 22.9 months; log-rank test, *P* = 0.02). The ECM-high tumors were characterized by elevated epithelial–mesenchymal transition and glycolytic activities, and low oxidative phosphorylation, E2F, and DNA repair pathway activities. This study offers novel insights into the protein network underlying pancreatic cancer opening up for proteome precision medicine development.

**Significance:**

Pancreatic cancer lacks reliable biomarkers for prognostication and treatment of patients. We analyzed the proteome of pancreatic tumors, nonmalignant tissues of the pancreas and PDX-derived cell lines, and identified proteins that discriminate between patients with good and poor survival. The proteomics data also unraveled potential novel drug targets.

## Introduction

Pancreatic cancer is one of the most lethal malignancies worldwide with a 5-year survival rate of approximately 8% (1). Broad disparity in patient survival and response to therapy are evident but due to a lack of reliable biomarkers of diagnosis, prognosis, and response to therapy, the patients receive a “one size fits all” standard of care. Comprehensive characterization of the pancreatic cancer genome has revealed extensive molecular heterogeneity demanding biomarker-driven individualized therapy for patients with pancreatic cancer (2–4). Unfortunately, alterations in *KRAS*, *CDKN2A, TP53*, and *SMAD4* dominating the mutational landscape of the pancreatic tumors are currently undruggable (4). At the transcriptomic level, multiple studies have been conducted defining similar yet discrepant subgroups of disease with distinct biology and patient outcome (3, 5–7). The studies converge to two broad classes of tumor-specific subtypes; the well differentiated Classical subtype and the poorly differentiated Basal-like subtype. In addition, based on stromal features, the tumors are divided into at least two subtypes; Activated and Normal stromal subtypes (6–8). However, a complete consensus is yet to be reached, and the clinical utility of the transcriptomic subtypes remains undetermined. Also, targeted therapies and immunotherapy effective in other cancer types have failed to show an effect in patients with pancreatic cancer (9–11). It is thus apparent that pancreatic cancer biology is inadequately understood on the basis of only genomics and transcriptomics studies warranting characterization of the disease at a more functional level such as proteomics. Because of the technical difficulty associated with quantification of the full proteome, comprehensive characterization of the pancreatic cancer proteome is limited. Hence most questions regarding active protein networks and their clinical implications in pancreatic cancer remain unanswered. Because most studies show a weak correlation between the mRNA molecule and its respective protein product, proteomic analyses have the potential to reveal actionable targets not detectable at the RNA level.

In this study, we describe in-depth quantitative mass spectrometry–based proteomics data and investigate proteomic changes associated with pancreatic cancer. By employing Weighted Gene Co-expression Network Analysis (WGCNA) (12, 13), we have organized the pancreatic cancer proteome into biologically and clinically meaningful protein modules. Altogether, we provide an improved contextual understanding of the molecular basis of pancreatic cancer that can be therapeutically exploited.

## Materials and Methods

### Patients and Samples

In total, 42 patients with pancreatic ductal adenocarcinoma (PDAC) and five patients with chronic pancreatitis consecutively selected from the “Thematic Research Area Pancreatic Cancer” biobank at Oslo University Hospital were included. This study, linked to the specific biobank, was approved by the Regional Ethics Committee (REK 2015/738) and an institutional review board, and conducted in accordance with WMA Declaration of Helsinki. The patients included in this study were admitted to Oslo University Hospital between 2008 and 2011 for pancreatectomy due to suspicion of pancreatic malignancy. Written informed consent was obtained from all patients prior to collection of biospecimens and associated clinical information. One patient identified with metastasis at the time of surgery, one patient who received a nonstandard treatment line and one patient from which we had access to a patient-derived xenograft (PDX)-derived cell line only (and not tumor sample) were excluded from the survival analyses. The last follow-up date for these patients was July 2, 2019.

**FIGURE 1:**
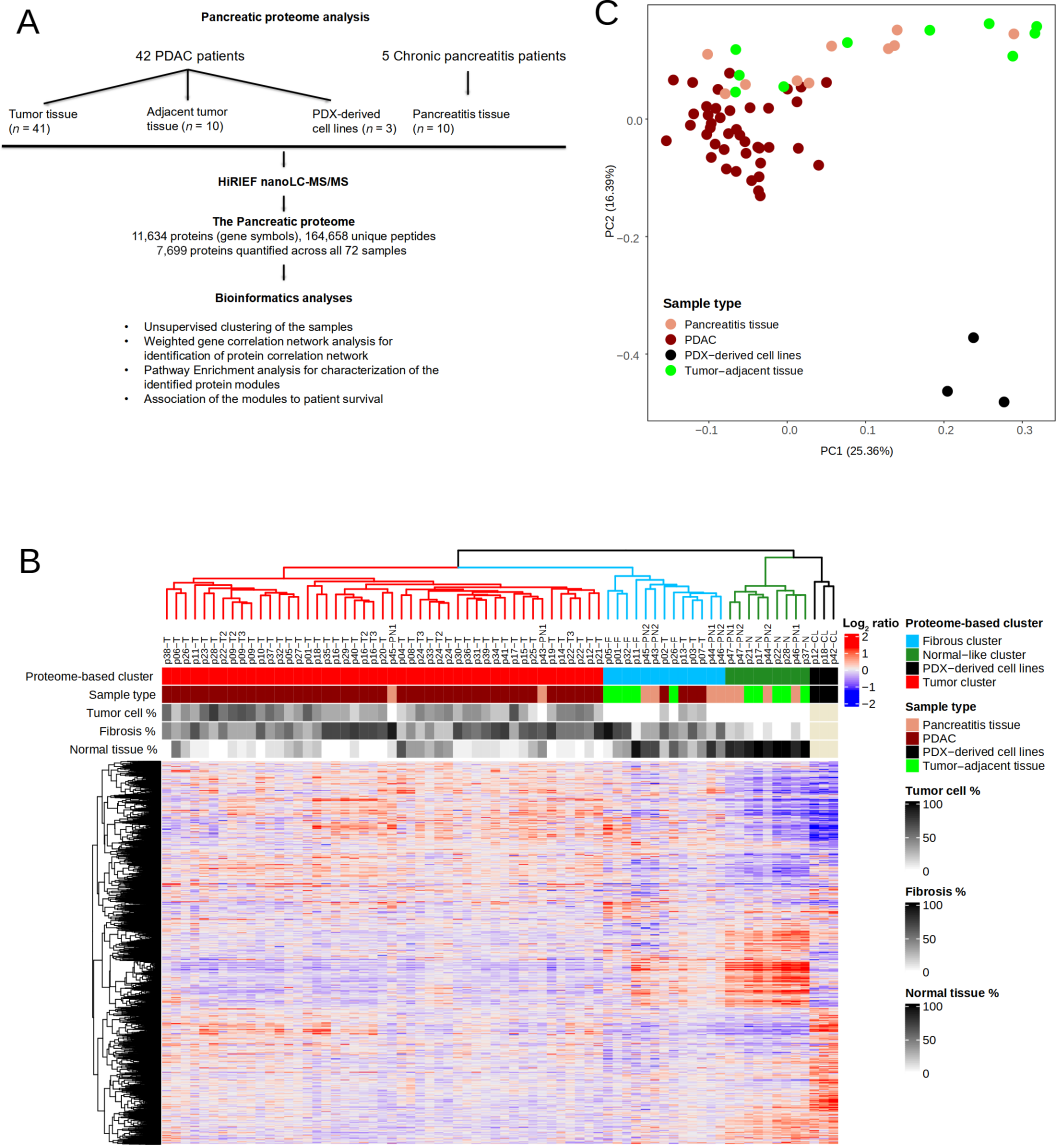
The pancreas proteome. **A,** The pancreas proteome analyses workflow. **B,** Hierarchical clustering of the proteins mapping to 7,699 gene symbols quantified in all 72 samples. **C,** Principal component analysis plot of the proteome data. Each dot represents a sample and each color represents the type of sample.

We quantified the proteome in a total of 72 samples (Fig. 1A; Supplementary Table S1). The samples from the 42 patients with pancreatic cancer included tumor tissue (*n* = 49; one sample each from 37 patients and three samples each from different tumor regions from four patients), tumor-adjacent tissue taken within a distance of 0.5–1.0 cm from the tumor (*n* = 10) and PDX-derived cell lines (*n* = 3). For the five patients with pancreatitis, two fresh frozen tissue samples from each (*n* = 10) were analyzed.

### Histopathologic Evaluation of Samples

Histopathologic evaluation of hematoxylin and eosin (H&E)-stained tissue sections was performed by an expert pathologist. All the samples were evaluated for the presence and proportions of malignant cells, fibrous tissue, and normal/other pancreatic tissue (Supplementary Table S2). Tissue areas with malignant cells formed the basis for tumor cellularity assessment. Areas with dense fibrosis including inflammatory regions were considered as fibrous tissue. Tissue areas with intact lobuli, normal-appearing acini and ducts, as well as small intestinal mucosa, smooth muscle wall, adipose tissues, peripheral nerves, blood and lymph vessels were considered as normal/other pancreatic tissue. None of the ten tumor-adjacent tissues contained malignant cells. Of these ten samples, six had normal pancreatic tissue morphology (intact lobuli, acinis, and ducts) while the other four had dense fibrotic tissue. The pancreatitis samples were found to be composed of varying proportions of normal pancreatic tissue, fibrotic stroma, and inflammatory tissue. The variation was also observed in the samples from the same patients.

### HiRIEF-nanoLC-MS/MS–based Proteomics

The tissue samples were lysed in 4% SDS and prepared for MS analysis using a modified version of the spin filter-aided sample preparation protocol. Tryptic peptides were Tandem Mass Tag (TMT) 10plex labeled and fractionated using immobilized pH gradient-isoelectric focusing (IPG-IEF) on a pH 3–10 strip (14). IPG-IEF Peptide fractions were separated using a 3000 RSLC-nano system and analyzed using a Thermo Scientific Q Exactive. MSGF + Percolator in the Galaxy platform was used to match MS spectra to the Ensembl 90 human protein database (15, 16). A detailed description can be found in the Supplementary Methods file.

Protein identifications were limited to 1% protein FDR (17). Protein quantification was performed using the TMT tag with a pool of all samples as denominator, and each tumor was normalized to its median ratio. The median Peptide spectrum matches (PSM) TMT reporter ratio from peptides unique to a gene symbol was used for quantification. The protein ratios were log_2_ transformed and each protein was further normalized to mean ratio within each TMT set. The normalized protein ratios are denoted as protein abundance or protein expression levels in figures and text.

### WGCNA

We used WGCNA to construct the coexpression network (12, 13). Briefly, a correlation matrix of all pair-wise correlations of proteins across the samples was generated and subsequently transformed into a weighted adjacency matrix by raising the correlation coefficients up to a soft threshold power β, to achieve a degree distribution that fits the hypothesis of scale-free network (12). The adjacency matrix was then used to build the Topological Overlap Matrix (TOM) that takes into account the topological similarity*,* that is*,* the similarity between two proteins, based on their coexpression relationships with all other proteins in the network. The proteins were then hierarchically clustered on the basis of the topological overlap dissimilarity measure (1 – TOM) using a dynamic tree-cutting algorithm (18) to generate coexpression modules.

Coexpression networks were constructed on the proteomics data from PDAC, pancreatitis and tumor-adjacent tissue samples (*n* = 61), and on data representing the PDAC only (*n* = 41). The top 25% most variable proteins (*n* = 1925) across each dataset were used in the analyses in both cases (Supplementary Table S3). The resulting weighted adjacency matrix achieved a degree distribution consistent with the topological structure of scale-free networks at β value of 10 and 12 for the datasets, respectively (Supplementary Fig. S1). The analysis was performed in R using the “blockwiseModules” function in R package WGCNA (13, 19). For both the “networkType” and “TOMtype” arguments in the function, the value “signed” was chosen. For the argument “corType”, the value “bicor” was chosen and the “minModuleSize” was set to 50. For all other arguments, default settings were used.

### Module Membership and Hub Genes

For each module, the Module Eigenproteins (ME), defined as the first principal component of the expression matrix of the corresponding module was calculated. This ME value per definition explains the maximal possible variability for all proteins within the module and is equivalent to the weighted average expression profile, and hence, is used as the module representative. Module membership (kME) indicating the proximity of a protein to a module was determined by calculating the Pearson correlation between each individual protein and the ME. The proteins with the highest kME are those with high network connectivity in a particular module, and are referred to as “intramodular hub proteins”.

### PPI Network Construction

Known protein–protein interaction networks for the proteins of the identified modules were constructed using the STRING database v.11 (20). The active interaction sources used were Experiments, Databases, Coexpression, and Cooccurrence. The minimum required interaction score was set to a high confidence (0.700) as specified by the string database.

### Transcriptomic Subtyping

Matched transcriptomic data were available for 38 of the 41 PDAC samples from our previous study (21). The tumors were assigned a tumor and a stromal transcriptomic subtype (Supplementary Table S2) as defined by Moffitt and colleagues (6). Subtype assignment was performed by consensus clustering of the samples (all 49 samples with transcriptomics data) on 50 tumor genes and 48 stromal genes as defined by Moffitt and colleagues (6). Consensus clustering consisted of 1,000 iterations of k-means clustering, with 80% of features and items subsampled and 20% hold-out at each iteration, followed by hierarchical clustering of the consensus matrix with complete linkage.

### Survival Analyses

The prognostic value of each of the modules was assessed in two steps. First, consensus clustering as described in section “Transcriptomic subtyping” was performed on the top 50 proteins with the highest correlation to the Module Eigenproteins. Second, a log-rank test was used to test for survival differences between the obtained consensus clusters. The Kaplan–Meier estimator was used to estimate overall survival (OS) curves, defined as the time from surgery to the time of death from any cause.

### Other Statistical Analyses

All statistical analyses were performed in R version 4.0.3 (22). Associations between variables were assessed with Fisher exact tests, *t* tests and Kruskal–Wallis tests as appropriate. Differential protein expression analysis between two groups were performed using the R package DEqMS (23). Enrichr webtool was used for pathway enrichment analysis on the protein members of the identified module (24) and the pathway activity in the protein subgroups was assessed using the R package Qusage (25).

### Data Availability Statement

The MS data have been deposited in the ProteomeXchange database under the accession code PXD025120. The transcriptomic dataset referenced in the study is available in the Gene Expression Omnibus repository with accession ID-GSE60979.

## Results

### The Pancreatic Cancer Proteome

The study cohort included tumor tissues, tumor adjacent tissues and PDX-derived cell lines from patients with pancreatic cancer. In addition, we also included and analyzed samples from patients with chronic pancreatitis (Supplementary Table S1). This allowed us to investigate proteome differences between pancreatic tumors, normal and stromal compartments, as well as pancreatitis tissues. The proteome profiles of the 72 samples were obtained by quantitative MS-based proteomics. The method identified 164,658 unique peptides corresponding to 11,634 genes with a 1% protein FDR providing a comprehensive coverage of a cell proteome. The proteome was quantified using a gene symbol centric approach (denoted protein henceforth), and all downstream analyses were performed on 7,699 proteins quantified across all the 72 samples. The quantified proteins had a median of eight unique peptides/protein and 12 peptides spectrum matches that were used for quantification (Fig. 1A; Supplementary Fig. S2). To obtain an unbiased view of the quantified proteome, we performed hierarchical clustering of the 72 samples on the 7,699 proteins. The analysis grouped the samples into four distinct proteome-based clusters (Fig. 1B). Cluster one (red) consisted mainly of pancreatic ductal adenocarcinomas (PDAC; *n* = 37) and two pancreatitis samples; the cluster is henceforth referred to as the tumor cluster. The second cluster (light blue) consisted of four PDAC, five tumor-adjacent tissues, and three pancreatitis tissue samples. All but one sample in this cluster was histopathologically characterized to have an abundance of fibrous tissue; hence the cluster is referred to as the fibrous cluster. The third cluster (green) consisted of five adjacent normal tissues and four pancreatitis samples, all with predominant normal pancreatic tissue, and it is henceforth referred to as the normal-like cluster. The fourth cluster (black) consisted of the three PDX-derived cell lines, which showed a clearly altered proteome compared with tumor samples as also demonstrated by principal component analysis (PCA; Fig. 1C). We further annotated the PCA plot by the sample batches to rule out TMT sets (sample batches) as a confounding variable (Supplementary Fig. S3). The biological replicates of PDAC samples showed significant correlation at the global protein levels, hence one representative sample per patient was used in further analyses (Supplementary Fig. S4).

### Network Analysis Identified Five Biologically Meaningful Modules

To unfold the protein phenotypic differences between the protein-based clusters representing different morphologic and biological states of the pancreatic tissue, we analyzed the proteomics data by WGCNA. The PDX-derived cell lines were excluded from the analysis as they exhibited a distinctly different protein phenotype. We also performed WGCNA on the proteomic data from the PDAC only (*n* = 41) to specifically capture the variation in protein biology underlying pancreatic cancer. We found a significant overlap between the most variable proteins (*n* = 1515) between the datasets, and consequently identified similar modules (Supplementary Fig. S5). Therefore, only the protein modules obtained from the WGCNA performed by using all the pancreatic tissue samples (*n* = 61) is described.

**FIGURE 2:**
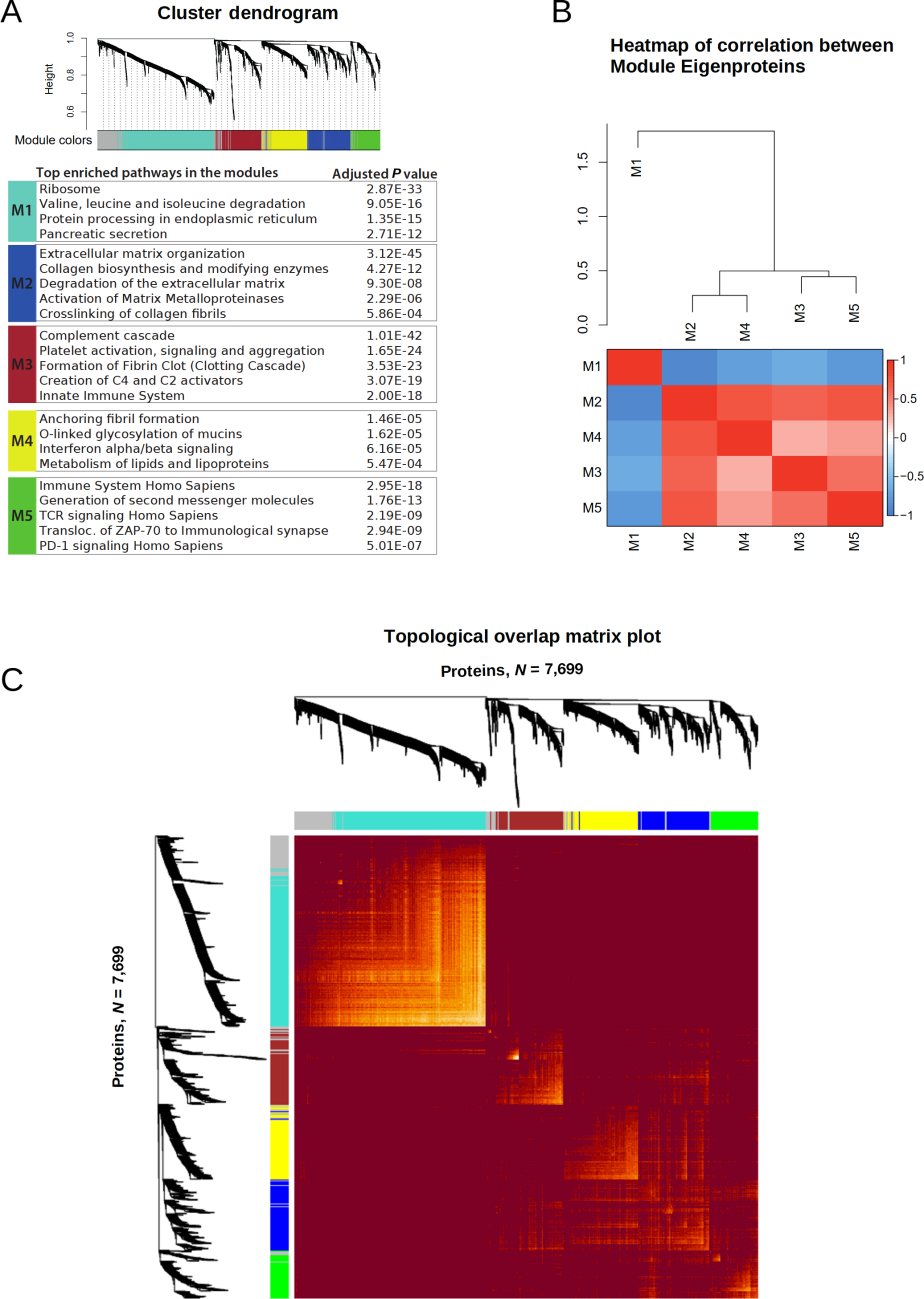
Coexpressed protein modules identified by WGCNA. **A,** Cluster dendrogram showing the corresponding protein dendrograms and module assignment of the proteins. Representative enrichments in each module are presented below (see Supplementary Tables S5–S9 for all enrichments). **B,** Heatmap of correlation between Module Eigenproteins illustrating (dis)similarities between modules. **C,** Heatmap of Topological Overlap Matrix illustrating higher intra-connectedness between proteins of the same modules. Rows and columns correspond to proteins, dark colors represent low topological overlap (low intra-connectedness), and progressively lighter orange and yellow colors represent higher topological overlap (high intra-connectedness).

Five protein modules, M1–M5, comprising of 635, 293, 279, 274, and 188 proteins, respectively, were identified (Fig. 2A; Supplementary Table S4). Of the 1,925 proteins used in the analysis, 256 remained unassigned to any module and were therefore excluded from further analyses (M0). We investigated the intermodular relationship by hierarchical clustering of the correlations between the Module Eigenproteins (MEs; defined in Materials and Methods). M1 was least similar to any of the other identified modules (Fig. 2B). A positive association was observed between modules M2–M5, with M2 showing the highest positive correlation to M4, and M3 to M5.

The proteins within a given module showed high topological overlap indicating high intra-connectedness as illustrated in Fig. 2C. A PCA on the quantitative proteomics data used in the WGCNA analysis (*n* = 1925) further confirmed that the proteins sharing a module have similar quantitative protein profile (Supplementary Fig. S6). We then examined the modules for known protein–protein interaction using the String database (20). For each identified module, significantly more interactions were evident for the proteins within a module than what would have been expected for a random set of proteins of similar size (Supplementary Figs. S7–S11). We further characterized the modules by functional enrichment analysis where each module was found to be associated with specific biological functions. In summary, M1 was enriched with proteins related to normal exocrine functions of the pancreas, M2 represented extracellular matrix organization, maintenance, and degradation, M3 was associated with the complement and coagulation cascade, M4 was associated with tumor cell signature, and M5 was found to be enriched with immune system components (Fig. 2A; Supplementary Tables S5–S9).

**FIGURE 3:**
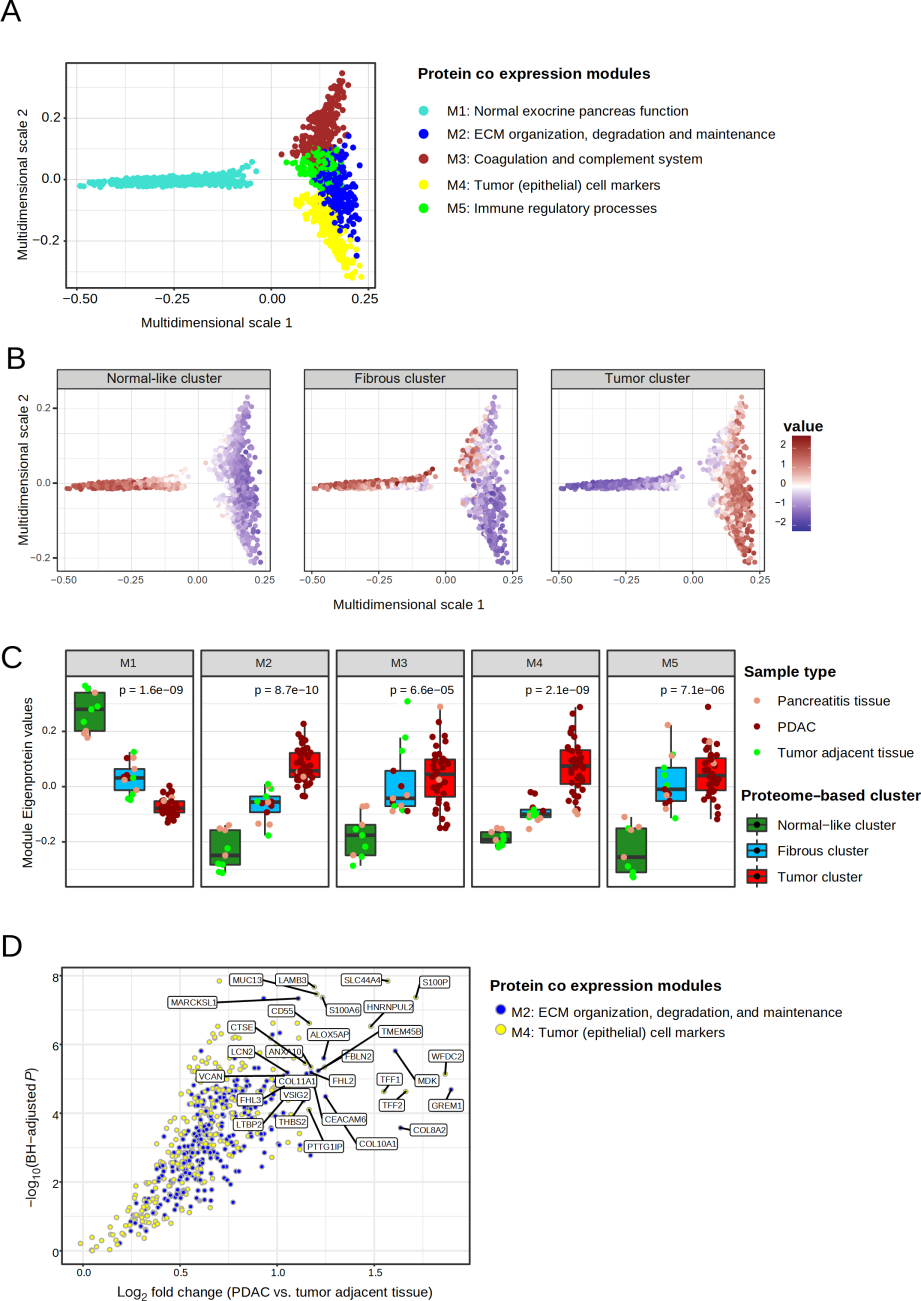
MDS of the TOM dissimilarity matrix and association of the protein modules to different tissues of the pancreas. **A,** MDS plot where each dot denotes a protein and the color represents the module the protein belongs to. **B,** MDS plot stratified by proteome-based clusters. Each protein is colored by the average expression in the proteome-based clusters (red, high expression; purple, low expression). **C,** Association of the coexpression protein modules (M1-M5) to the proteome-based clusters. Color of the dot indicate sample type. The *P* value denotes significance by Kruskal–Wallis test. **D,** Volcano plot illustrating differential protein abundances in PDAC versus tumor adjacent tissue. The log_2_ fold change in protein abundance is represented on the *x*-axis and Benjamini–Hochberg adjusted *P* values (on negative log scale) is shown on the *y*-axis. Each dot represents a protein and is colored by the coexpression module that the protein belongs to. The 15 most significant proteins of M2 and M4 are labeled.

Next, we performed multidimensional scaling (MDS) of the topology overlap matrix (TOM) representing the coexpression network; the MDS plot is portrayed in Fig. 3A.

By overlaying the coexpression network by average protein levels in the normal-like, fibrous, and tumor clusters, we observed distinct module wise difference in protein abundances between the proteome-based clusters (Fig. 3B). Consistently, the MEs were found to be significantly differentially expressed between the three proteome-based clusters, illustrating the connection between the clustering and module analyses (Fig. 3C; Kruskal–Wallis test; *P* < 0.001). The MEs, with the exception of ME4, showed a larger variation in the sample groups when using the pathology-based tissue labels, implying that the redefined proteome-based sample groups are biologically more similar (Supplementary Fig. S12A). Taken together, the M1 module showed high specificity to the normal-like cluster. The M2 and M4 module proteins were specifically abundant in the tumor cluster, while the M3 and M5 modules were equally abundant in both the fibrous and the tumor clusters.

We further examined the expression of the MEs in the cell lines with respect to the proteome-based clusters. We found absence/low levels of the M1, M2, M3, and M5 proteins in the cell lines implying that these are not expressed in the tumor epithelium. Levels of the M4 proteins were notably high in the cell lines confirming specificity of the module to tumor cells (Supplementary Fig. S12B).

### Normal Pancreatic Function is Highly Correlated with Proteins of the Cellular Translational Machinery

The normal-like cluster featured higher abundance of M1 proteins and low levels of M2–M5 proteins (Fig. 3B and C). Functional enrichment analysis of the proteins in the M1 module showed overrepresentation of proteins governing the exocrine function of the pancreas (e.g., AMY2A, AMY2B, PNLIP, CPA1/2, CELA2A/2B, and CTRC). In addition, metabolism of branched chain amino acids (e.g., BCAT1, BCAT2, BCKDHB) and the protein translational machinery including structural subunits of ribosomes (RPSs and RPLs), several EIFs and proteins involved in intracellular trafficking and protein processing in the Endoplasmic Reticulum (SRPs and SSRs) were among the top hits in the enrichment analysis (Supplementary Table S5). We further found that the levels of cytosolic ribosomal proteins were lower in the tumor cluster and the PDX-derived cell lines compared with the normal-like cluster. The mitochondrial ribosomal protein levels were lower in the tumor cluster than in the normal-like samples but notably higher in the PDX-derived cell lines (Supplementary Fig. S13), presumably reflecting the different growth conditions *in vitro* and *in vivo*.

### Pancreatic Tumors are Characterized by High Abundance of Tumor Epithelial Cell Proteins and Extracellular Matrix Proteins

The levels of M2 and M4 proteins were significantly higher in the tumor cluster compared with both the normal-like and fibrous clusters (Fig. 3C). The M2 module was highly enriched for proteins involved in ECM organization, maintenance, and degradation (Supplementary Table S6). It included several actin filament-associated regulatory proteins involved in remodeling of the cytoskeleton such as the collagens (e.g., COL3A1, COL5A2, and COL10A1), filamins (e.g., FLNA and FLNC), and microfibril-associated proteins (e.g., MFAP4, MFAP5). The module also represented members of the lysyl oxidase protein family (e.g., LOX, LOXL1 and LOXL2), matrix metalloproteases (MMP), and the metallopeptidase inhibitors (TIMP1, TIMP2, and TIMP3) known to be associated with tumorigenesis as well as cadherins (e.g., CDH11, CDH12), carcinoembryonic antigen-related cell adhesion molecules (CEACAMS) and FN1 that are important for cell–cell and cell–matrix interaction.

The M4 module included established and candidate oncoproteins such as MET, CHEK1, and BCAS1 and the cell proliferation marker MKI67. O-linked glycosylated proteins such as the MUC gene family, GALNT5 and GALNT7, as well as epithelial keratin markers (e.g., KRT6A, KRT7, and KRT10) were members of this module. The module also contained proteins with pivotal roles in antiviral activities including MX1, ISG15, OAS1, OAS2, and IFIT1 (Supplementary Table S8).

A majority of the M2 and M4 proteins showed a significant higher abundance in PDAC compared with the tumor-adjacent tissues (Fig. 3D; Supplementary Tables S10A and B) indicating specificity of these proteins to PDAC. We further observed that 18 of 21 proteins listed to be upregulated in PDAC compared with adjacent tumor tissue/normal ducts in a recent study (26) were members of M2 (*n* = 7) and M4 (*n* = 11; Supplementary Table S11). Of the overlapping set of proteins, the levels of WFDC2, S100P, CD55, MDK, THBS2, and MFAP2 were more than 2-fold higher in PDAC compared with tumor-adjacent tissue in our data (Fig. 3D).

### Protein-based Tumor and Fibrous Clusters have Similar Immune Profiles

The M3 and M5 modules, as mentioned earlier, were equally abundant in the tumor and fibrous clusters (Fig. 3C). Proteins in the M3 module (Supplementary Table S7) mainly belonged to the complement system, and hence components of the innate immune system, and the coagulation cascade including platelet activation signaling and fibrin clotting components. It included platelet degranulation proteins, represented by several members of the Serpin superfamily of proteins (e.g., SERPINA1, SERPINB7, SERPIND1), complement factors (e.g., C1S, C4A, C6, and C9), and a large number of immunoglobulins (e.g., IGHG2, IGHD and IGHA2). In addition, cholesterol homeostasis and lipid transporter proteins (e.g., APOA1, APOE, APOC3, APOM and LCAT, CETP, PLTP) were also represented. The M5 module contained proteins associated with immune regulatory processes (Supplementary Table S9). Both modules consisted of a wide range of proteins essential for T-cell development and T-cell receptor signaling, members of MHC class I and II complexes as well as several B- and T cell–specific marker molecules (e.g., CD3D, CD48, CD84 and CD247). Moreover, the intracellular lymphocyte-specific enzymes p56LCK and ZAP70, and adapter proteins SKAP1 and SKAP2 important for immune cell functions were also represented.

### A Novel ECM Protein Signature Predicts Patient Survival

**FIGURE 4:**
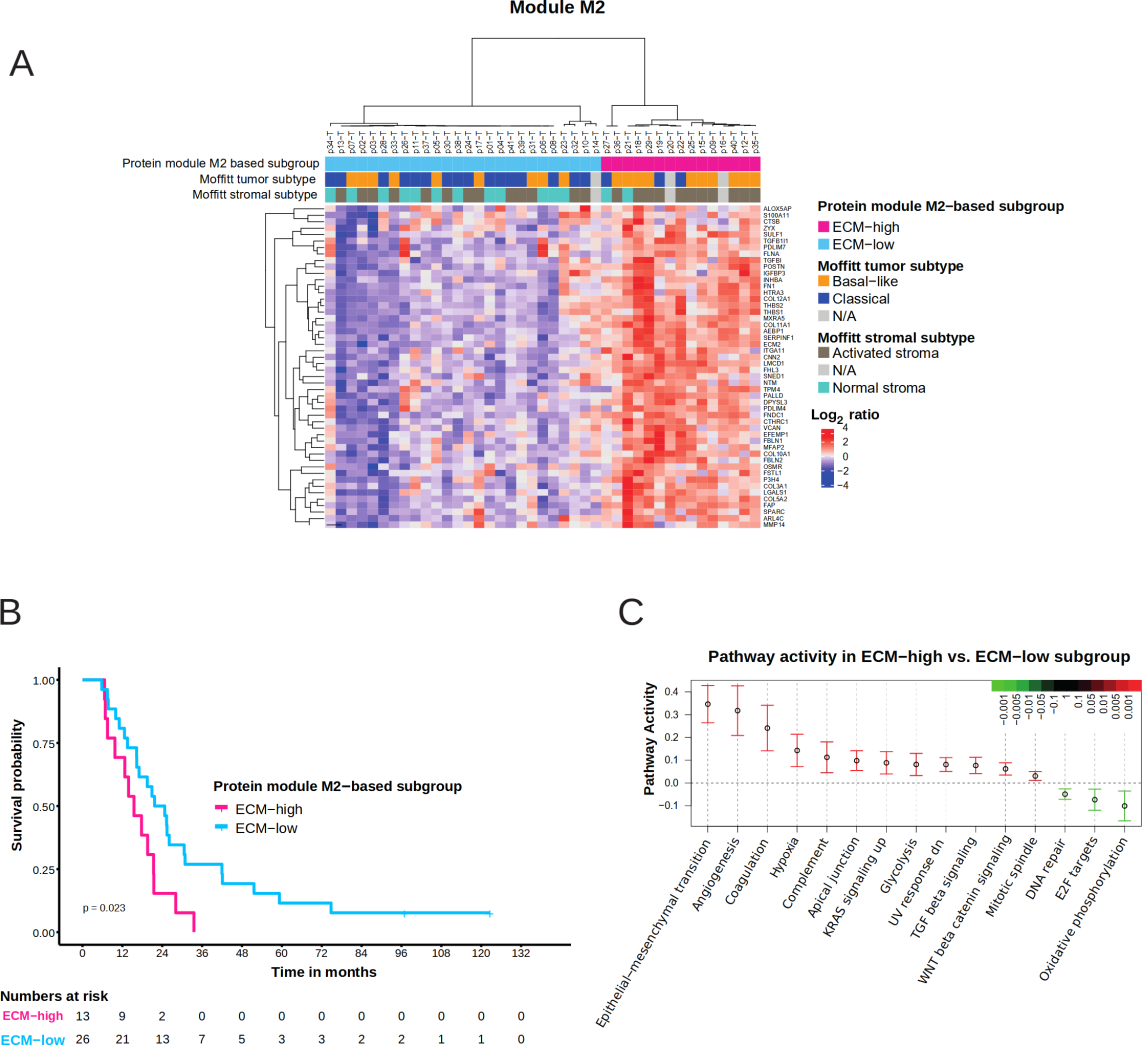
M2 module with enriched pathways, relation to Moffitt transcriptomic subtypes and patient outcome. **A,** Consensus clustered heatmap of top 50 proteins (based on correlation to Module Eigenprotein) of module M2 enriched for ECM proteins; patients get stratified into two clusters. **B,** Kaplan–Meier curve showing overall survival trends in the protein-based ECM subgroups **C.** Enriched pathways in the ECM-high versus ECM-low subtypes.

Consensus clustering of the tumor samples using the top 50 proteins with the highest correlation to the ME of the M2 module stratified the patients into two distinct subgroups which were labeled as ECM-high and ECM-low (Fig. 4A, Supplementary Fig. S14; Supplementary Table S2). The ECM-high and ECM-low subgroups showed significantly different overall survival, where the patient group with the lower levels of the ECM proteins survived longer (Fig. 4B, median survival months: 15.3 *vs.* 22.9 months; log-rank test, *P* = 0.02). A similar exercise performed on the other identified modules did not reveal any association to patient prognosis.

The ECM-based subgroups were further analyzed for differences in pathway activity (Fig. 4C). Epithelial–mesenchymal transition (EMT) pathway was found to be highly upregulated in the ECM-high group. Several EMT markers and key EMT players such as FN1, VIM, MMP2, ZEB1, LMCD1 were represented. Furthermore, the ECM-high tumors were featured by high glycolytic activity and TGFβ signaling, and low DNA repair, E2F targets and oxidative phosphorylation.

We further investigated the association of the novel protein subtypes to both tumor and stromal transcriptomic subtypes as defined by Moffitt and colleagues (6). We found that most of the samples classified as the Normal stroma subtype (12/14) and the Classical tumor subtype (16/19) belonged to the ECM-low subgroup (Fig 4A). A larger ambiguity was observed for the Activated stroma and the Basal-like tumor subtypes, with an almost equal distribution between the ECM-high and ECM-low subgroups. The proteome phenotype is hence distinctly different from the transcriptome phenotype. The transcriptome-based subtypes did not show any significant association with patient prognosis in our cohort (Supplementary Figs. S15A and B). Apart from the ECM-based subgroups, none of the standard clinical parameters were found to be associated with patient outcome in univariate cox regression analysis in this cohort. The ECM-based subgroups also showed a strong trend toward an independent association to patient outcome (*P* = 0.067) in a multivariate Cox regression model analysis including pathologic differentiation grade and the tumor-specific transcriptomic subtypes (variables showing most significant trend of association with patient survival in univariate analysis; Supplementary Table S12).

### Pancreatic Tumors and Pancreatitis Proteome—Differences and Similarities

**FIGURE 5:**
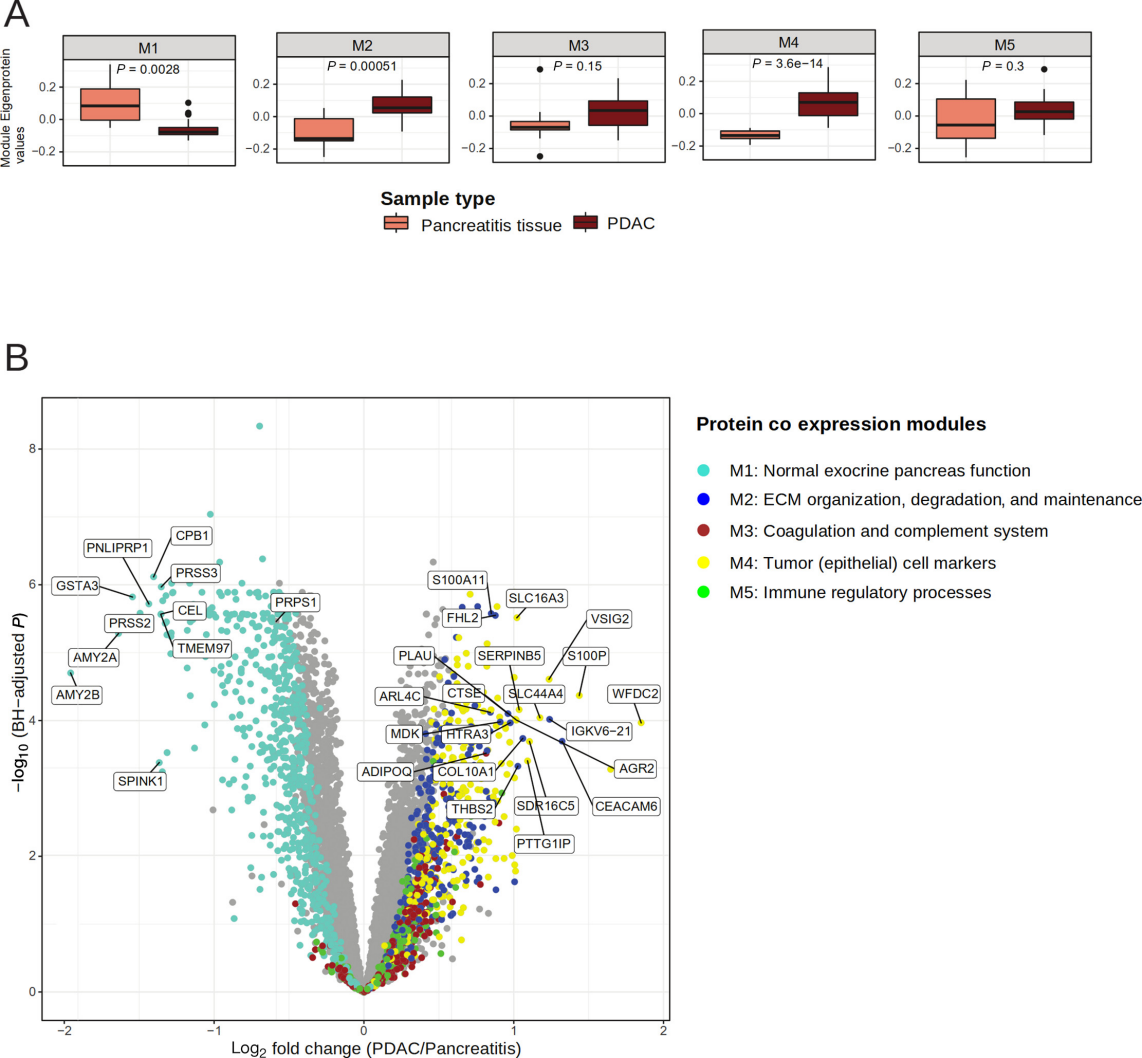
Differences between the PDAC and the pancreatitis proteome. **A,** Boxplots illustrating the expression of Module Eigenproteins in PDAC versus pancreatitis samples. **B,** Volcano plot illustrating differential protein abundances in PDAC versus pancreatitis samples. The fold change (log_2_) in protein abundance is represented on the *x*-axis and Benjamini–Hochberg adjusted *P* values (on negative log scale) is on the *y*-axis. Each dot denotes a protein, colored by the coexpression module the protein belongs to. The top 10 significant proteins of M1, M2, and M4 are labeled.

Biomarkers that can discriminate between patients with PDAC and chronic pancreatitis are of great clinical interest. Hence, we evaluated whether the identified protein modules could differentiate the two diseases. The M1 and M4 modules associated with normal pancreatic function and the tumor epithelium, respectively, showed a significant differential expression between the two diseases (Fig. 5A; *P* < 0.003). This difference may be explained by the difference in cell composition.

Interestingly, among the modules representing stroma, the ECM module (M2) was significantly elevated in the PDAC compared with the pancreatitis samples. We did not observe any significant differences in expression levels of the M3 module proteins associated with the complement and coagulation cascade or the M5 module proteins associated with immune regulatory processes. We further analyzed differential protein expression between PDAC and pancreatitis samples using the DeqMS tool (23). The analysis further confirmed that proteins belonging to modules M1, M2, and M4 were significantly differentially expressed between PDAC and pancreatitis (Fig. 5B).

### Intramodular Hub Proteins as Potential Drug Repurposing Targets

**FIGURE 6:**
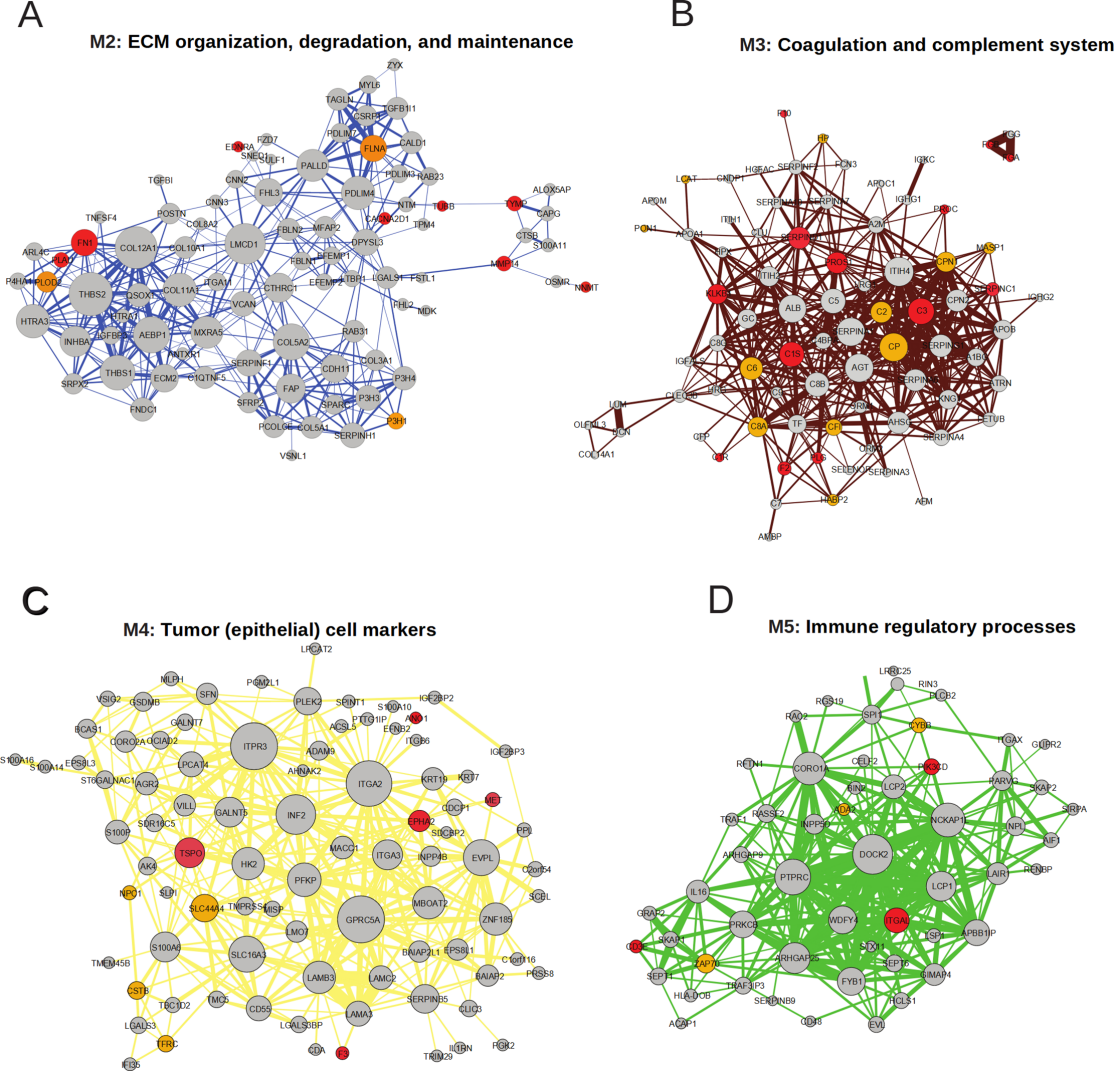
Protein network of the protein modules including proteins with intercorrelations > 0.3 and correlation to Module Eigenprotein > 0.7. The size of the nodes represents degrees, and thickness of the edges represents edge weight. Targets of FDA-approved drugs (all indications) within each module are marked red and potential targets are marked orange. **A,** Module M2. **B,** Module M3. **C,** Module M4. **D,** Module M5. String protein networks corresponding to M2–M5 are represented in Supplementary Figs. S8–S11, respectively. M1 is excluded from the analysis due its specific association to the normal pancreas function and its low expression in the tumor tissue which makes it less relevant for drug targets.

Protein levels in modules M2–M5 were significantly higher in the pancreatic tumors compared with adjacent normal pancreatic tissues implying possible important roles in the tumors and a less critical role in the normal functions of the pancreas. This property makes the proteins in modules M2–M5, specifically the hub proteins potential drug target candidates. To identify potential drug targets in the modules, we cross-referenced the lists of M2–M5 proteins with (i) targets of FDA-approved drugs and (ii) potential targets. Several proteins with known roles in tumorigenesis such as FN1, TSPO, CD3E, and ZAP70 were present in the modules (Fig. 6), which may be explored further as novel drug targets in pancreatic cancer.

## Discussion

In this study, we have characterized the global proteome of PDAC and various nonmalignant tissues of the pancreas with high analytic depth. Prior to protein quantification by the LC/MS-MS technique, the complexity of the sample proteome was reduced using the peptide level HiRIEF separation technique (14), yielding high-quality quantitative proteome data. In an unsupervised hierarchical clustering, the PDAC, adjacent pancreatic tissue, pancreatitis samples, and PDX-derived cell lines clustered into four distinct groups. The clustering seemed to be driven largely by the proportions of normal tissue, fibrotic tissue, and tumor cells in the samples. This was particularly distinct for the adjacent pancreatic tissues, where half of the samples showing normal pancreas morphology had a protein profile very different from the other half, which contained abundant tissue fibrosis. These observations, while biologically important, also advocate for the robustness of the data as well as the key results presented herein.

The network based WGCNA organized the global proteome into several modules that were associated with specific biological processes. In accordance with pancreatic exocrine function, proteins for digestive enzymes encompassing amylases, lipases, and proteases were all grouped into one module (M1) and were significantly overexpressed in the normal pancreatic tissue compared with the other tissues. It should be noted that the transcripts encoded by pancreatic tissue-specific genes comprise up to 68% of the total mRNA pool. This is in contrast to almost all other tissue types examined, where genes with “housekeeping functions” dominate the mRNA pool (27). Consequently, even small proportions of normal tissue residuals may contribute to molecular signals that can be incorrectly interpreted as disease heterogeneity. This directly impedes studies directed at defining disease subtypes, as has been implied earlier, in that the exocrine-like subtype of PDAC is attributed to normal tissue contribution and not tumor biology (6).

The M1 module with high protein levels in normal samples was also highly enriched for components of the protein translation machinery. Low abundance of the digestive enzymes in the pancreatic cancer samples mainly comprising epithelial cells and stromal tissue, which are not specialized in protein secretion is as expected and reported earlier (6, 28). Reduced or altered expression of the protein translation machinery in pancreatic cancer compared with normal pancreatic tissue is, however, sparsely reported. Our finding of decreased protein translation system in pancreatic cancer supports and substantiates the observation that several ribosomal subunits are significantly downregulated in the PDAC in a small-scale protein-based study (29). This is in line with protein synthesis rate being more than 2.5-fold lower in pancreatic tumor compared with healthy pancreatic tissue (30). Sparse reports on this molecular change, which is highly evident at the protein level is likely due to large-scale molecular studies in pancreatic cancer being almost exclusively mRNA-based, in addition to the low mRNA–protein correlation observed for the ribosomal proteins (31–33). The high rate of protein synthesis in the pancreas has been linked to the pronounced secretory protein production in the acinar cells (34), and ribosomal protein expression is found to vary between tissues and even cell types (35). The difference in expression levels of the protein translation machinery between tumor and normal pancreatic tissue may in part reflect the paucity of the acinar cells in the bulk tumors. It may also be a consequence of the metabolic reprogramming that follows pancreatic cancer tumor development. Regardless of the mechanism behind reduced protein synthesis, and the emerging moonlighting roles of individual components of the protein translational machinery in oncogenesis (35), our observations point to the fact that the intracellular demand for protein synthesis in normal pancreatic tissue exceeds that of the proliferating malignant cells of pancreas. This needs to be taken into account when considering the protein synthesis machinery and key regulators of translational control as therapeutic targets in pancreatic cancer.

The ECM-associated M2 module and the tumor cell marker–associated M4 module, significantly distinguished PDAC from both tumor adjacent tissue and pancreatitis tissue. Most of the proteins belonging to these modules were significantly elevated in the PDAC compared with the nonmalignant tissues. It therefore provides a very useful resource for biomarker validation and development of novel diagnostic and potentially predictive analyses. Interestingly, the ECM module-based proteins discriminated between patients with PDAC with good and poor overall survival. The ECM-high tumors showed significantly elevated EMT pathway activity and poor patient prognosis. The two subgroups of tumors had also marked difference in metabolic programs. Glycolytic pathway activity was found to be high while oxidative phosphorylation was low in the ECM-high subgroup which may indicate altered metabolic activity and associated nutrient partitioning in these tumors. These observations are largely in line with findings of Cao and colleagues where they show that the poor-prognosis proteogenomic Basal-like subtype is enriched for EMT and glycolytic pathway signature among others (26). Together, these results suggest that the poor prognosis of the patients with ECM-high tumors may be attributed to the metastasis promoting role (36) as well as chemoresistance associated with EMT (37). Tumor targeting through abrogating metabolic programs/pathways, an approach that relies on differential metabolic programs between the tumors cells and its microenvironment (38) may benefit a selected patient population. Further experimental investigation is warranted to fully exploit the potential of the differential signaling pathways and associated proteins between ECM-low and ECM-high tumors as prognostic and predictive markers.

Extensive desmoplasia is a common hallmark feature of both PDAC and pancreatitis known to account for the markedly similarities in stromal features at both the histologic and the molecular level (39, 40). However, the two diseases are associated with differential prognosis and require suitable clinical management; hence markers that discriminate between PDAC and chronic pancreatitis are of great clinical importance. Interestingly, among the three protein modules representing the stromal microenvironment (M2, M3 and M5), the M2 (ECM module) was significantly downregulated in the pancreatitis samples compared with the PDAC. Of note is that although the immune related modules were not differentially expressed between PDAC and pancreatitis, the modules were significantly elevated in both of the diseases compared to tumor adjacent tissue. This is in agreement with the established role of inflammation in initiating and sustaining a stromal milieu favorable for the development of both diseases (41). Reports on lower expression of the ECM related proteins in pancreatitis compared with PDAC are sparse. Recently, in a comprehensive analysis of the stroma from progressive stages of PDAC and pancreatitis, PDAC represented the most fibrotic disease state, implying an increasing ECM complexity in the PDAC over the course of disease progression (42). Although the presence of neoplastic cells in the PDAC is a defining biological difference between the diseases, these often constitute as little as 10% of the total tumor volume and are less likely to be shed in the blood stream compared with the ECM-related proteins. Hence, the highly abundant ECM related protein markers, which we reveal here to be significantly overexpressed in PDAC, bears higher potential as biomarkers for the diagnosis of PDAC as well as differential diagnostics of PDAC and pancreatitis.

By analyzing the PDAC tumors together with diverse yet related biological samples of the pancreas including tumor and normal tissue, PDX-derived cell lines and pancreatitis tissue, we provide an enhanced contextual understanding of the active protein network underlying pancreatic cancer. Overall, this study provides a high-quality proteomic resource and lays the foundation for improved molecular classification of patients, and contributes to identification of novel drug targets for the successful treatment of patients with PDAC.

## Supplementary Material

Supplementary Methods SM1 Supplementary method describing HiRIEF-nanoLC-MS_MS based proteomics technology

Figures FS1-FS15 Supplementary figures

Table S1 Patients and samples overview

Table S2 Histological composition of the samples along with assigned transcriptomics and proteomics classifications for PDAC

Table S3 Top 25% most variable proteins used in WGCNA

Table S4 Proteins members of modules M1-M5

Table S5-S9 Enriched pathways in Module M1-M5

Table S10A and S10B Differential expression analysis of M2 and M4 proteins between PDAC and tumor adjacent tissue samples (Wilcoxon-rank sum test)

Table S11 Proteins (and the assigned module) identified in our dataset overlapping with the proteins with significantly higher abundance in PDAC tumor tissue compared to adjacent tissue/normal ducts in the study of Cao et al.2021

Table S12 Univariate cox regression analysis (left) including clinical, pathological, and molecular variables. Multivariate cox regression analysis (right) including the variables with significant or trending association with patient survival in the univariate analysis.
