# Supplementary material for: Proteome Analysis of Pancreatic Tumors Implicates Extracellular Matrix in Patient Outcome
Source: Cancer Res Commun. 2022 Jun 14;2(6):434–46. doi: 10.1158/2767-9764.CRC-21-0100 (PMC10010336; doi:10.1158/2767-9764.CRC-21-0100)
Supplement: Supplementary Methods SM1 — Supplementary method describing HiRIEF-nanoLC-MS_MS based proteomics technology [file crc-21-0100-s01.docx]

## HiRIEF-nanoLC-MS/MS based proteomics

### **Sample preparation for mass spectrometry.**

Tissue samples were lysed with 4% SDS, 25 mM HEPES, 1 mM DTT. Samples were prepared using a modified version of the spin filter aided sample preparation protocol[^1,2^](https://paperpile.com/c/7a7fum/RYDE+bzvC). Lysates were heated to 95°C for 5 min followed by sonication for 1 min and centrifugation at 14,000g for 15 min. The supernatant was mixed with 1 mM DTT, 8 M urea, 25 mM HEPES, pH 7.6 and transferred to a 10-kDa cut-off centrifugation filtering unit (Pall, Nanosep®), and centrifuged at 14,000g for 15 min. Proteins were alkylated by 50 mM iodoacetamide (IAA) in 8 M urea, 25 mM HEPES for 10 min. The proteins were then centrifuged at 14,000g for 15 min followed by 2 more additions and centrifugations with 8 M urea, 25 mM HEPES. Trypsin (Promega) in 250 mM urea, 50 mM HEPES was added to the cell lysate at a ratio of 1:50 trypsin:protein and incubated overnight at 37°C with gentle shaking. The filter units were centrifuged at 14,000g for 15 min followed by another centrifugation with MQ and the flow-through was collected. Peptides were labelled with TMT10plex reagent according to the manufacturer’s protocol (Thermo Scientific) and cleaned by a strata-X-C-cartridge (Phenomenex).

### **IPG-IEF of peptides.**

TMT labelled peptides were separated by immobilized pH gradient - isoelectric focusing (IPG-IEF) on pH 3-10 strips as described by Branca *et al* [*^1^*](https://paperpile.com/c/7a7fum/RYDE)*.* Peptides were extracted from the strips by a prototype liquid handling robot, supplied by GE Healthcare Bio-Sciences AB. A plastic device with 72 wells was put onto each strip and 50 µl of MQ was added to each well. After 30 minutes of incubation, the liquid was transferred to a 96 well plate and the extraction was repeated 2 more times. The extracted peptides were dried in speed vac for storage and dissolved in 3% acetonitrile (ACN), 0.1 % formic acid before MS analysis.

### **Q Exactive analysis.**

Before analysis on the Q Exactive (Thermo Fisher Scientific, San Jose, CA, USA), peptides were separated using an Ultimate 3000 RSLCnano system. Samples were trapped on an Acclaim PepMap nanotrap column (C18, 3 µm, 100Å, 75 µm x 20 mm), and separated on an Acclaim PepMap RSLC column (C18, 2 µm, 100Å, 75 µm x 50 cm), (Thermo Scientific). Peptides were separated using a gradient of A (5% DMSO, 0.1% FA) and B (90% ACN, 5% DMSO, 0.1% FA), ranging from 6 % to 37 % B in 30-90 min (depending on IPG-IEF fraction complexity) with a flow of 0.25 µl/min. The Q Exactive was operated in a data dependent manner, selecting top 10 precursors for fragmentation by HCD. The survey scan was performed at 70,000 resolution from 400-1600 m/z, with a max injection time of 100 ms and target of 1 x 10^6^ ions. For generation of HCD fragmentation spectra, a max ion injection time of 140 ms and AGC of 1 x 10^5^ were used before fragmentation at 30% normalized collision energy, 35,000 resolution. Precursors were isolated with a width of 2 m/z and put on the exclusion list for 70 s. Single and unassigned charge states were rejected from precursor selection.

### **Peptide and protein identification.**

Orbitrap raw MS/MS files were converted to mzML format using msConvert from the ProteoWizard tool suite. Spectra were then searched using MSGF+ (v10072)[^3^](https://paperpile.com/c/7a7fum/X0IA) and Percolator (v2.08) [^4^](https://paperpile.com/c/7a7fum/04Pl), where search results from 8 subsequent fractions were grouped for Percolator target/decoy analysis. All searches were done against the human protein subset of Ensembl 90 in the Galaxy platform[^5^](https://paperpile.com/c/7a7fum/W81b). MSGF+ settings included precursor mass tolerance of 10 ppm, fully-tryptic peptides, maximum peptide length of 50 amino acids and a maximum charge of 6. Fixed modifications were TMT10plex on lysines and peptide N-termini, and carbamidomethylation on cysteine residues, a variable modification was used for oxidation on methionine residues. Quantification of TMT10plex reporter ions was done using OpenMS project's IsobaricAnalyzer (v2.0). PSMs found at 1% FDR (false discovery rate) were used to infer gene identities. Protein false discovery rates were calculated using the picked-FDR method using gene symbols as protein groups and limited to 1% FDR[^6^](https://paperpile.com/c/7a7fum/FdvRX).

Protein quantification by TMT10plex reporter ions was calculated using TMT PSM ratios to the tissue sample pool and each tumor was normalized to its median ratio. The median PSM TMT reporter ratio from peptides unique to a gene symbol was used for quantification. The protein ratios were log2 transformed and each protein was further normalized to mean ratio within each TMT set. The normalized protein ratios are denoted as protein abundance or protein expression levels in figures and text.

References

1. [Branca, R. M. M. *et al.* HiRIEF LC-MS enables deep proteome coverage and unbiased proteogenomics. *Nat. Methods* **11**, 59–62 (2014).](http://paperpile.com/b/7a7fum/RYDE)

2. [Manza, L. L., Stamer, S. L., Ham, A.-J. L., Codreanu, S. G. & Liebler, D. C. Sample preparation and digestion for proteomic analyses using spin filters. *Proteomics* **5**, 1742–1745 (2005).](http://paperpile.com/b/7a7fum/bzvC)

3. [Kim, S. & Pevzner, P. A. MS-GF+ makes progress towards a universal database search tool for proteomics. *Nat. Commun.* **5**, 5277 (2014).](http://paperpile.com/b/7a7fum/X0IA)

4. [Granholm, V. *et al.* Fast and accurate database searches with MS-GF+Percolator. *J. Proteome Res.* **13**, 890–897 (2014).](http://paperpile.com/b/7a7fum/04Pl)

5. [Boekel, J. *et al.* Multi-omic data analysis using Galaxy. *Nat. Biotechnol.* **33**, 137–139 (2015).](http://paperpile.com/b/7a7fum/W81b)

6. [Savitski, M. M., Wilhelm, M., Hahne, H., Kuster, B. & Bantscheff, M. A Scalable Approach for Protein False Discovery Rate Estimation in Large Proteomic Data Sets. *Mol. Cell. Proteomics* **14**, 2394–2404 (2015).](http://paperpile.com/b/7a7fum/FdvRX)
