# Supplementary material for: Proteome Analysis of Pancreatic Tumors Implicates Extracellular Matrix in Patient Outcome
Source: Cancer Res Commun. 2022 Jun 14;2(6):434–46. doi: 10.1158/2767-9764.CRC-21-0100 (PMC10010336; doi:10.1158/2767-9764.CRC-21-0100)
Supplement: Figures FS1-FS15 — Supplementary figures [file crc-21-0100-s02.pdf]

Figure S1

A

Power determination for WGCNA in 61 samples

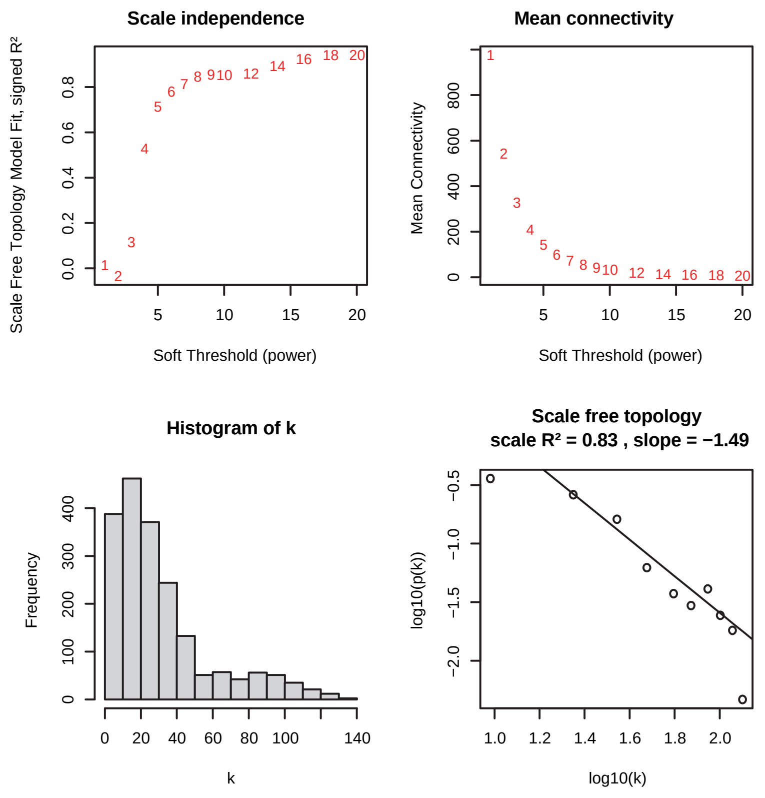

B

Power determination for WGCNA in 41 PDAC samples

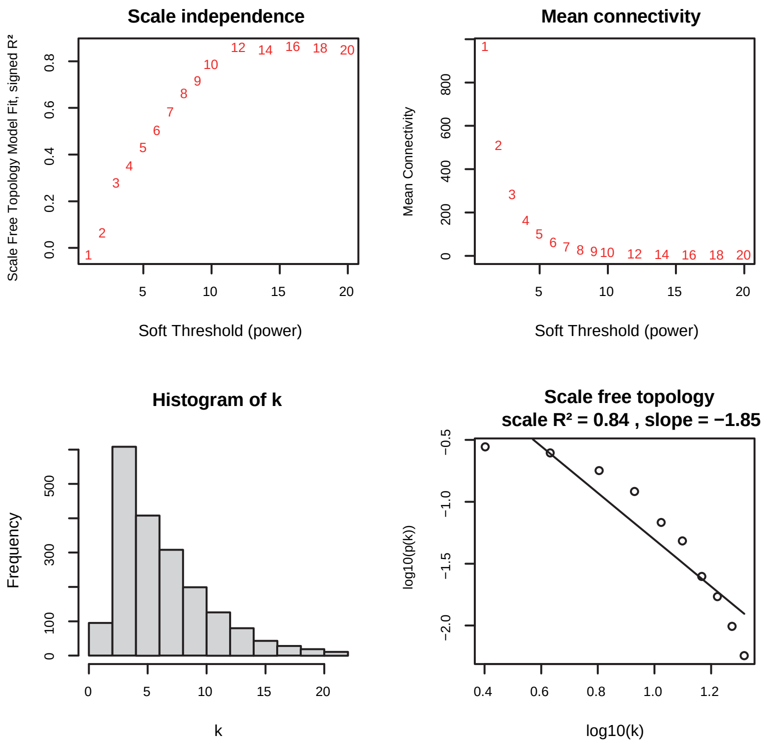

Figure S1. Power determination and connectivity distributions to show scale-free topology for **A**. WGCNA in 61 samples; **B.** WGCNA in 41 PDAC only. The frequency distribution of the connectivity (left) shows a large number of proteins with few connections and a small number of proteins with a large number of connections. The log-log plot shows an R2 (the scale-free topology index) of 0.83 and 0.84 for the two datasets which means that the network is following the scale-free topology criterion.

Figure S2

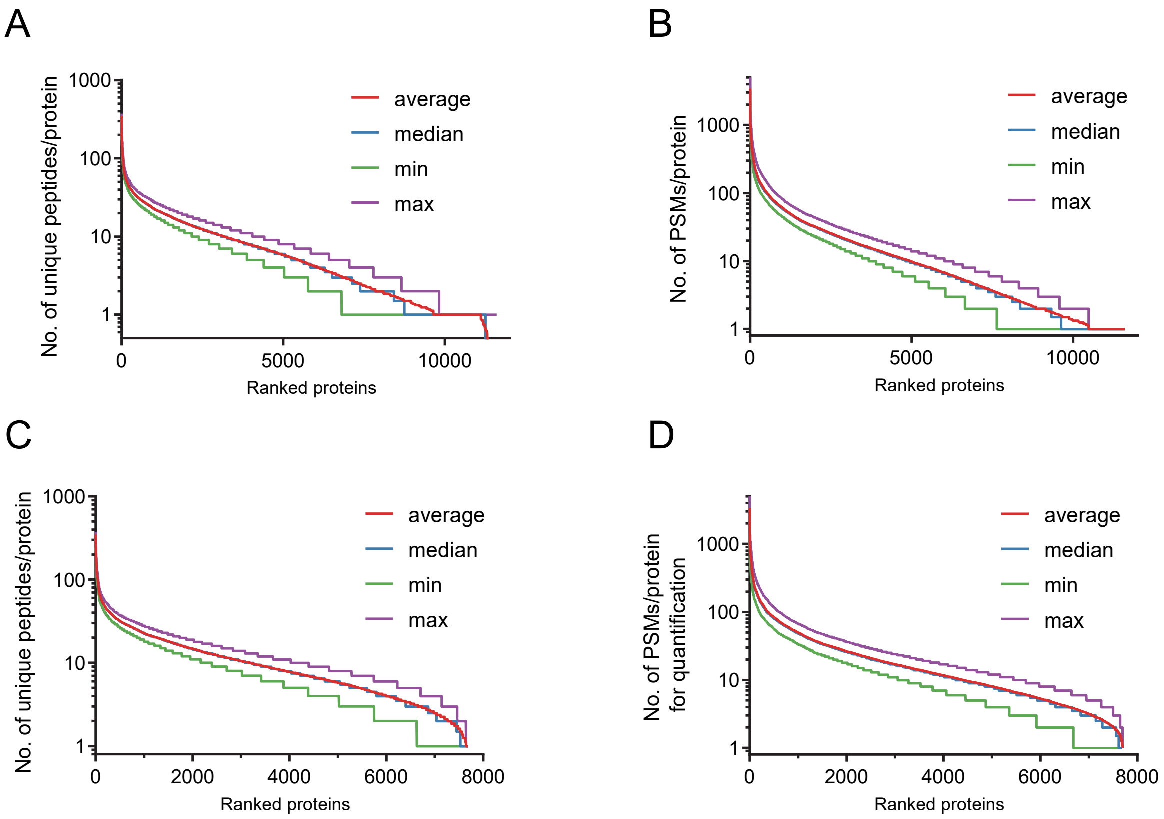

Figure S2. Robustness evaluation of the pancreas MS proteomics data (A, B). All 11634 identified proteins ranked by **A**. number of unique peptides per protein and **B.** number of peptide spectrum matches (PSMs) per protein for identification, across TMT10 set 1 to 8 (1% protein FDR). **C, D.** Ranking of the 7699 proteins with TMT quantification across all 8 TMT10 sets, based on (C) number of unique peptides per protein and (D) number of PSMs per protein for quantification. The protein quantification for this dataset is based on a median of 8 unique peptides/protein, and for 97% of the proteins, two or more PSMs per protein were used to calculate the TMT ratio. The average number of PSMs per protein used for protein quantification was 30 with 12 as median.

Figure S3

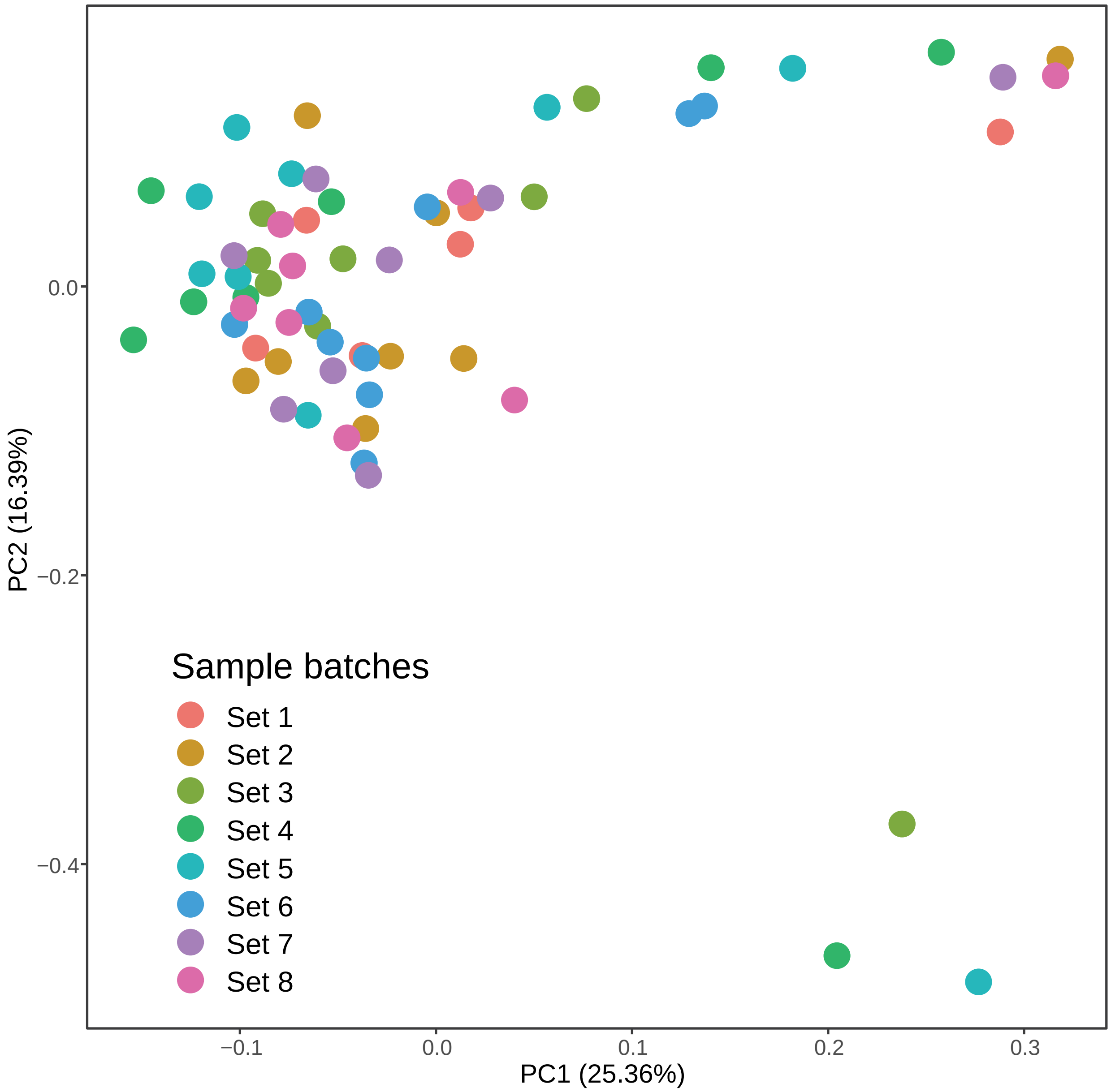

Figure S3. Principal component analysis plot of the proteome data. Each dot represents a sample and each color represents batch (TMT set) the samples were quantified in.

Figure S4

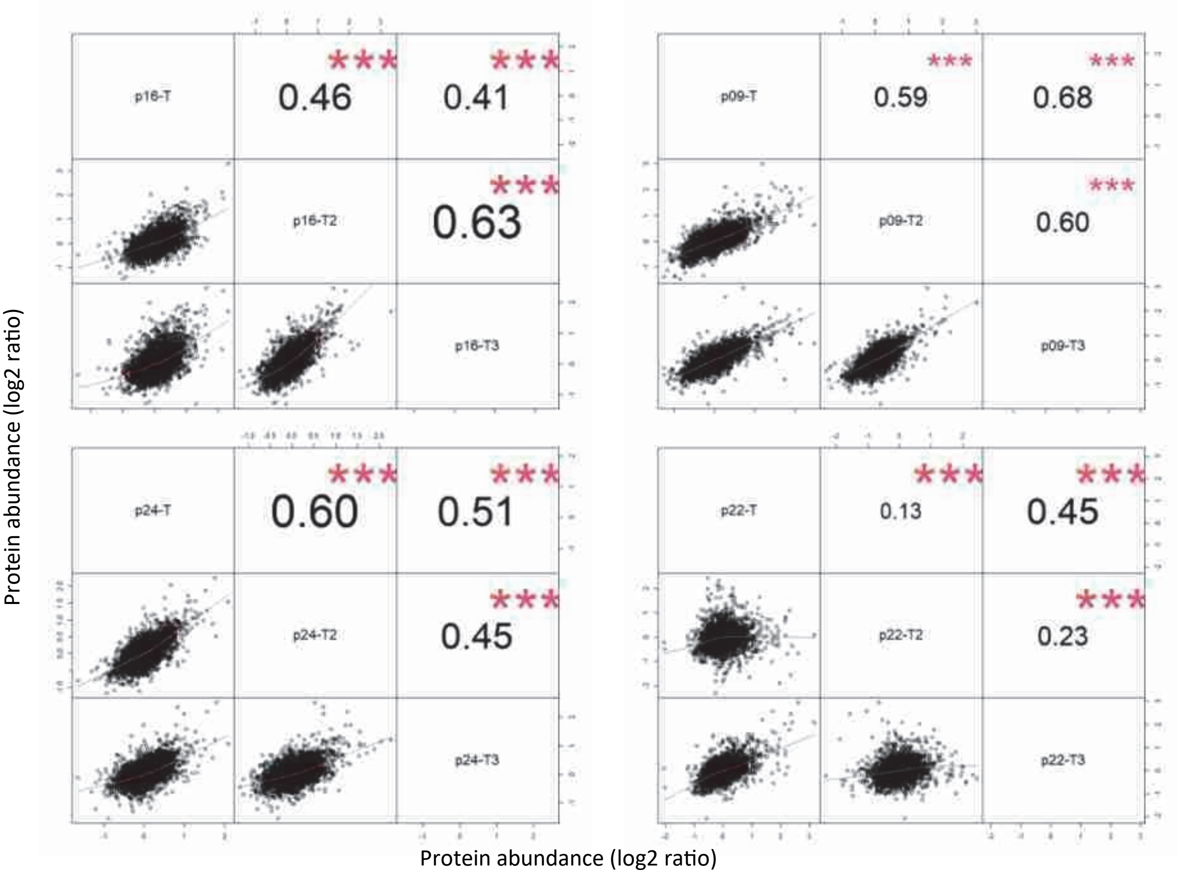

Figure S4. Scatterplot of protein levels in the biological replicates. The numbers indicate Spearman correlation coefficients, * denotes the p-value; * < 0.05; ** < 0.005; *** < 0.0005.

Figure S5

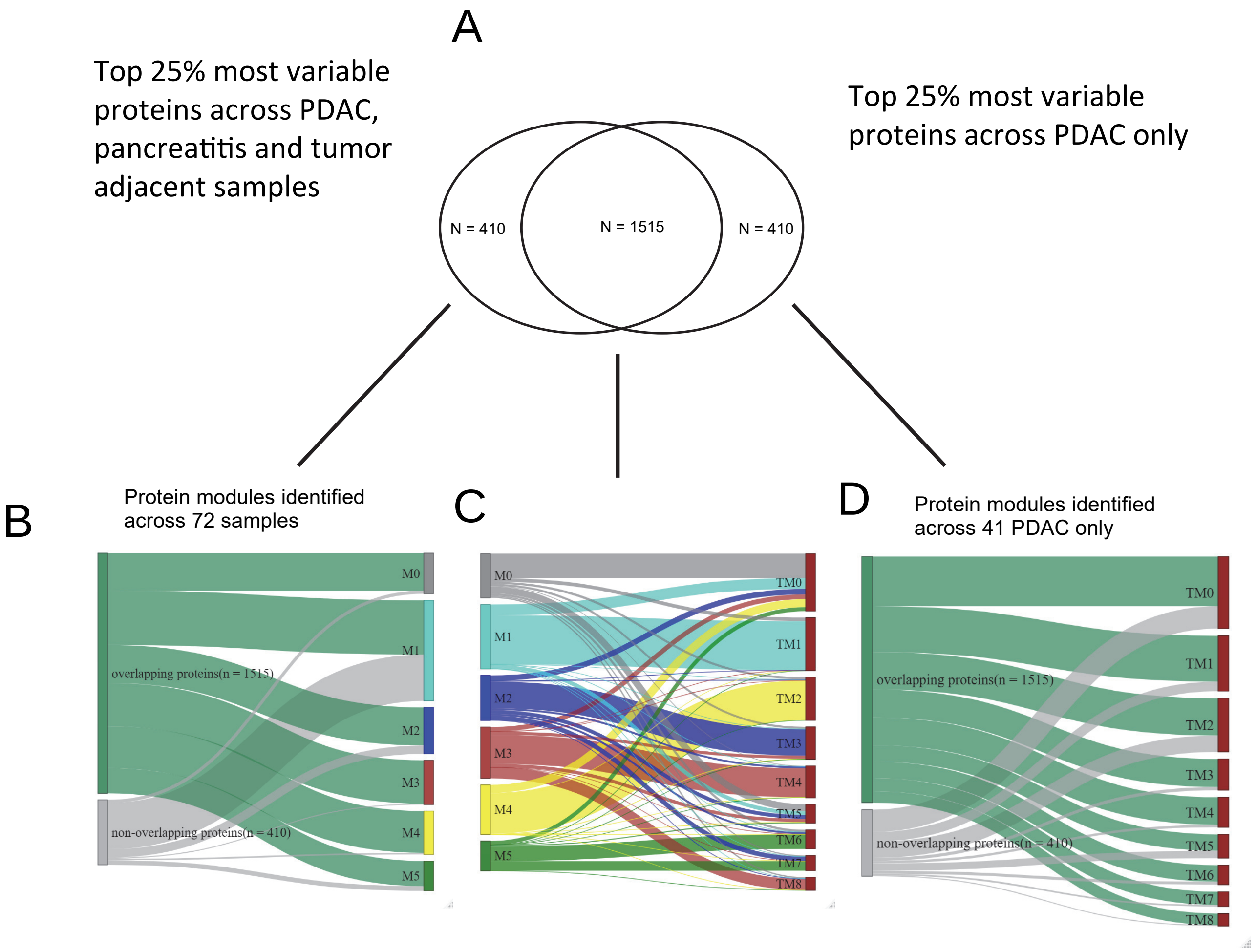

Figure S5. **A.** Venn diagram showing overlap of top 25% most variable proteins and the co-expression protein modules identified by WGCNA across all 72 samples and only in the PDAC; **B.** Sankey plot visualizing distribution of overlapping and non-overlapping proportions of proteins (Fig. S5A) into protein modules M0-M5 identified by WGCNA across all samples; **C.** Sankey plot visualizing distribution of members of M0-M5 into TM0-TM8 (proteins modules identified by WGCNA in the PDAC only); **D.** Sankey plot visualizing distribution of overlapping and non-overlapping proportions of proteins (Fig. S5A) into protein modules TM0-TM8 identified by WGCNA in the PDAC only.

Figure S6

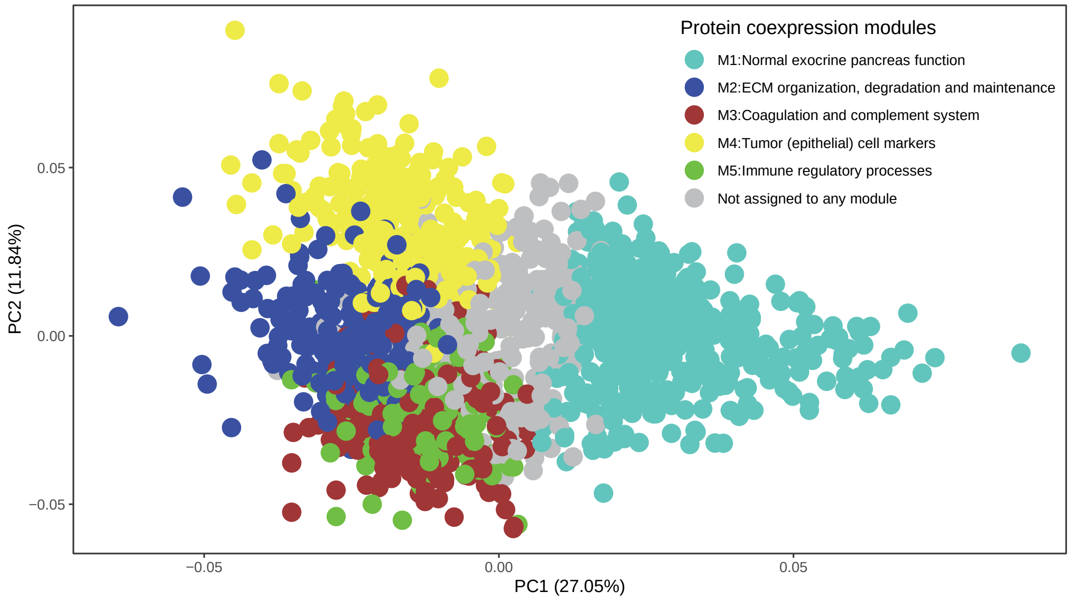

Figure S6. Principal component analysis plot of the proteome data. Each dot represents a protein and each color represents the module it belongs to.

Figure S7

Module M1: Normal pancreas function

Network stats

Number of nodes: 636
Number of edges: 3426
Average node degree: 10.8
Avg. local clustering coefficient: 0.454
Expected number of edges: 880
PPI enrichment p-value: < 1.0e-16

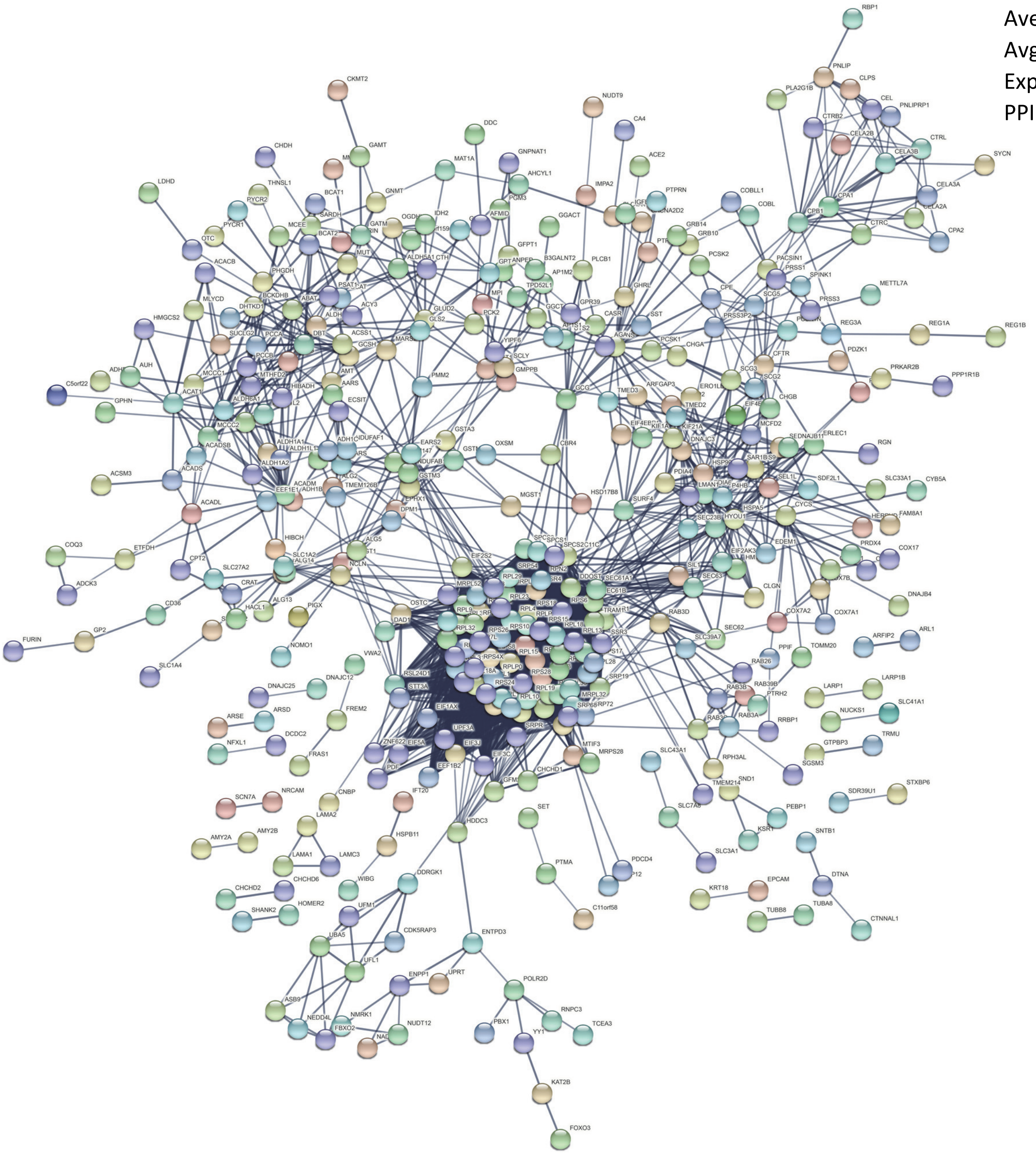

Figure S7. STRING Protein-protein interaction (PPI) network of module M1.The active interaction sources used were Experiments, Databases, co-expression and co-occurrence. The minimum required interaction score was set to a high confidence (0.700) as specified by the string database (https://string-db.org/stringdb.org).

Figure S8

Module M2: ECM organization, degradation and maintenance

Network stats

Number of nodes: 290
Number of edges: 609
Average node degree: 4.2
Avg. local clustering coefficient: 0.394
Expected number of edges: 104
PPI enrichment p-value: < 1.0e-16

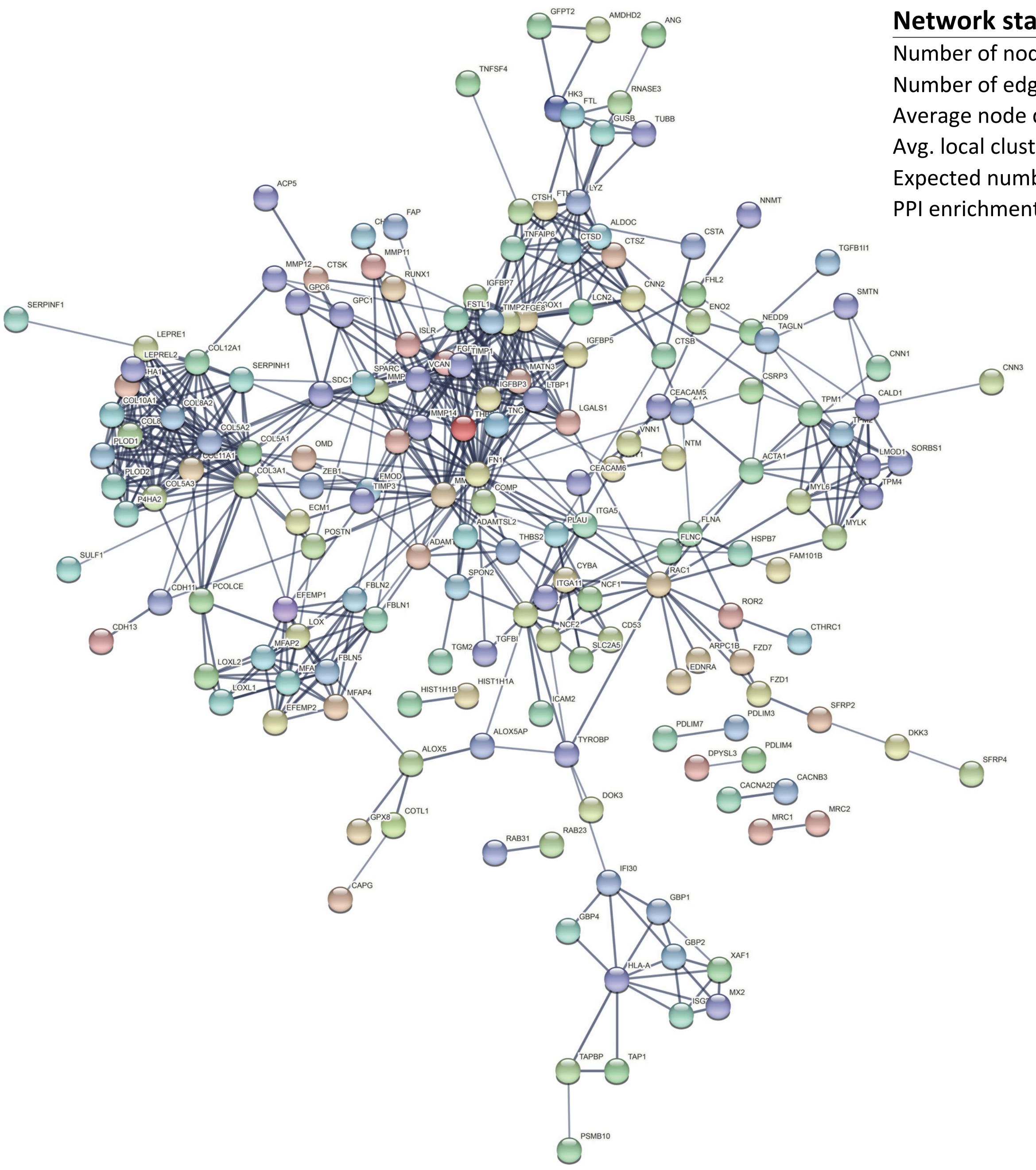

Figure S8. STRING Protein-protein interaction (PPI) network of module M2.The active interaction sources used were Experiments, Databases, co-expression and co-occurrence. The minimum required interaction score was set to a high confidence (0.700) as specified by the string database (https://string-db.org/stringdb.org).

Figure S9

Module M3: Coagulation and complement system

Network stats

Number of nodes: 248
Number of edges: 1309
Average node degree: 10.6
Avg. local clustering coefficient: 0.535
Expected number of edges: 147
PPI enrichment p-value: < 1.0e-16

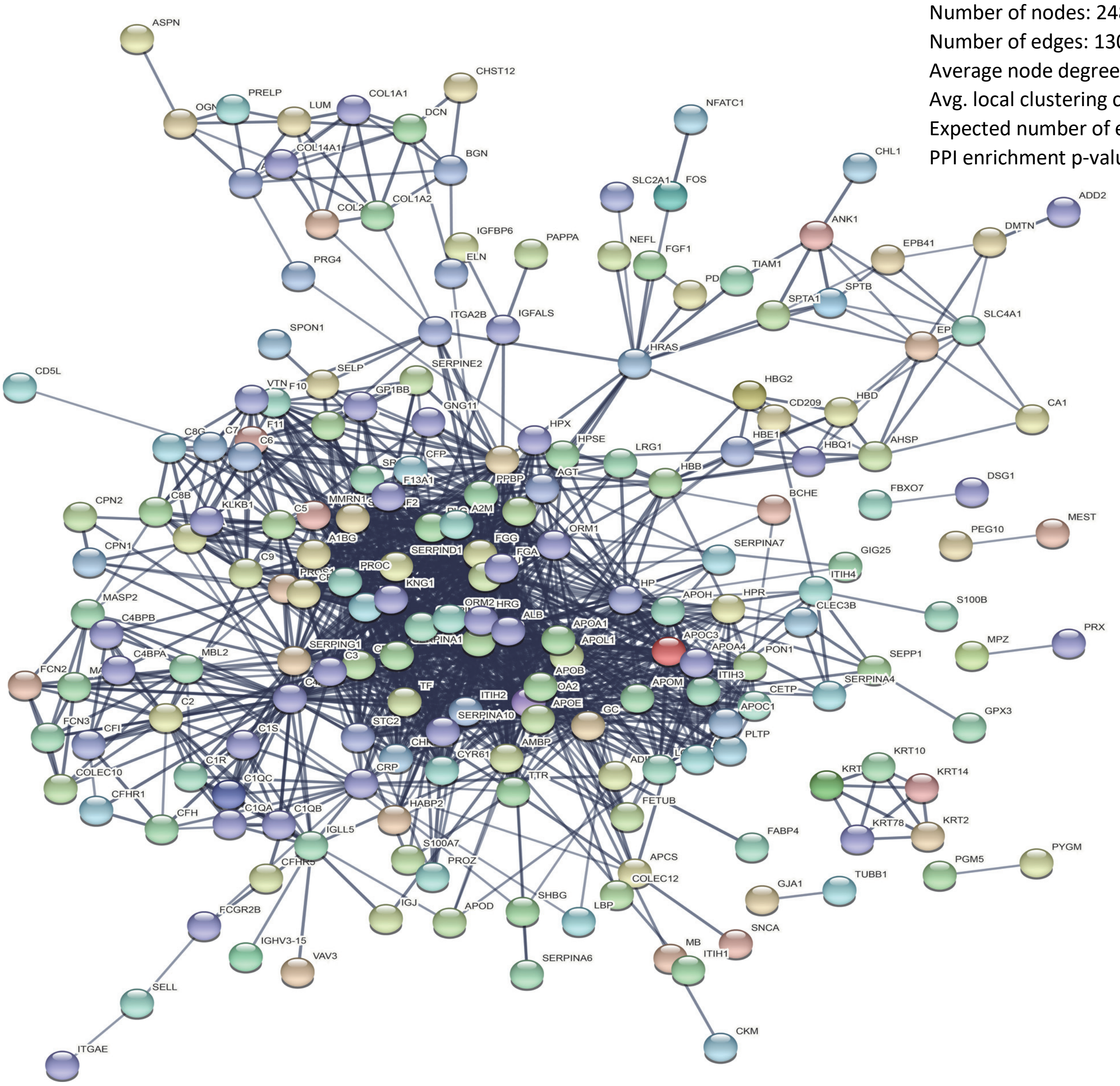

Figure S9. STRING Protein-protein interaction (PPI) network of module M3.The active interaction sources used were Experiments, Databases, co-expression and co-occurrence. The minimum required interaction score was set to a high confidence (0.700) as specified by the string database (https://string-db.org/stringdb.org).

Figure S10

Module M4: Tumor (epithelial) cell markers

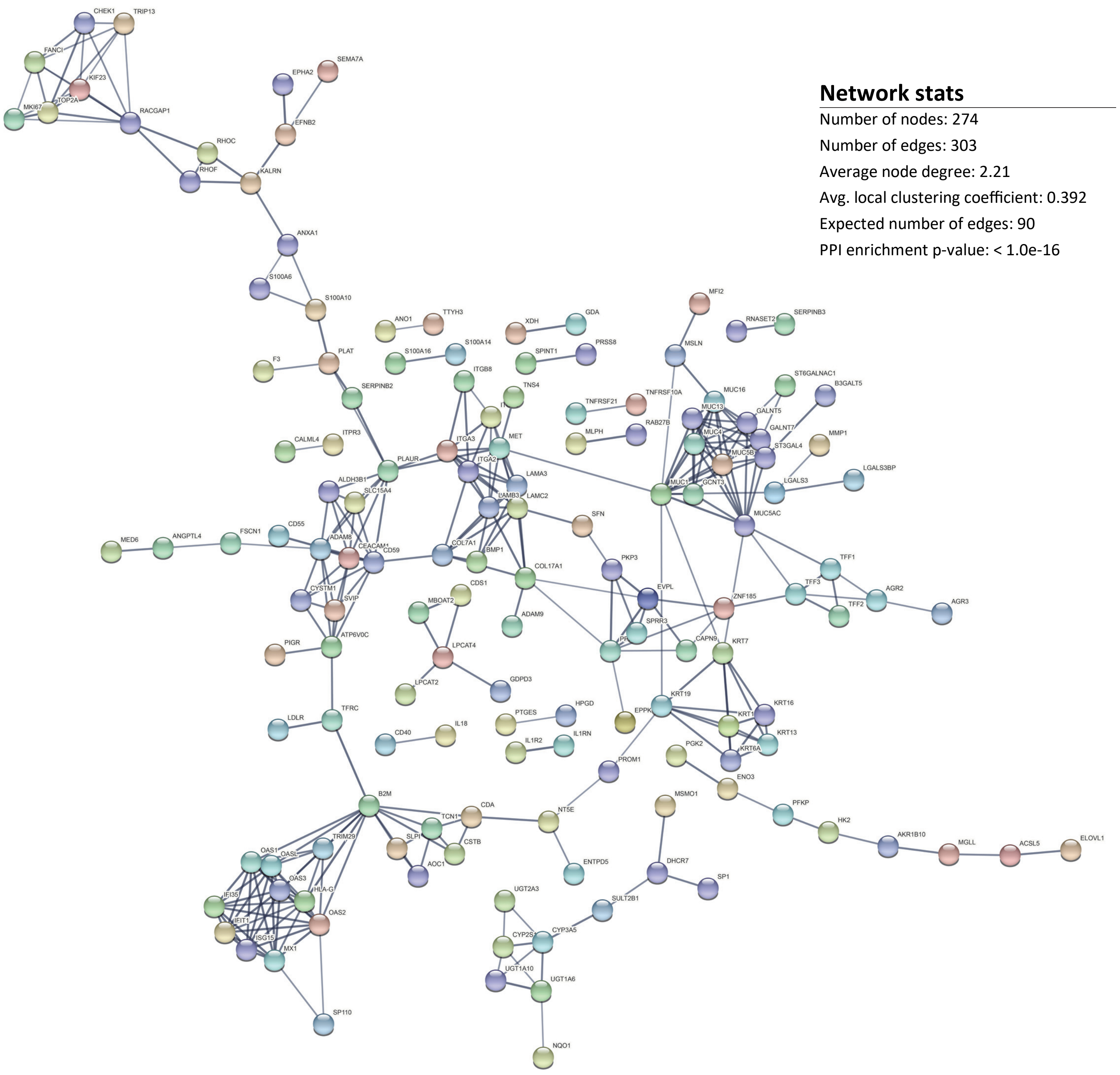

Figure S10. STRING Protein-protein interaction (PPI) network of module M4.The active interaction sources used were Experiments, Databases, co-expression and co-occurrence. The minimum required interaction score was set to a high confidence (0.700) as specified by the string database (https://string-db.org/stringdb.org).

Figure S11

Module M5: Immune regulatory processes

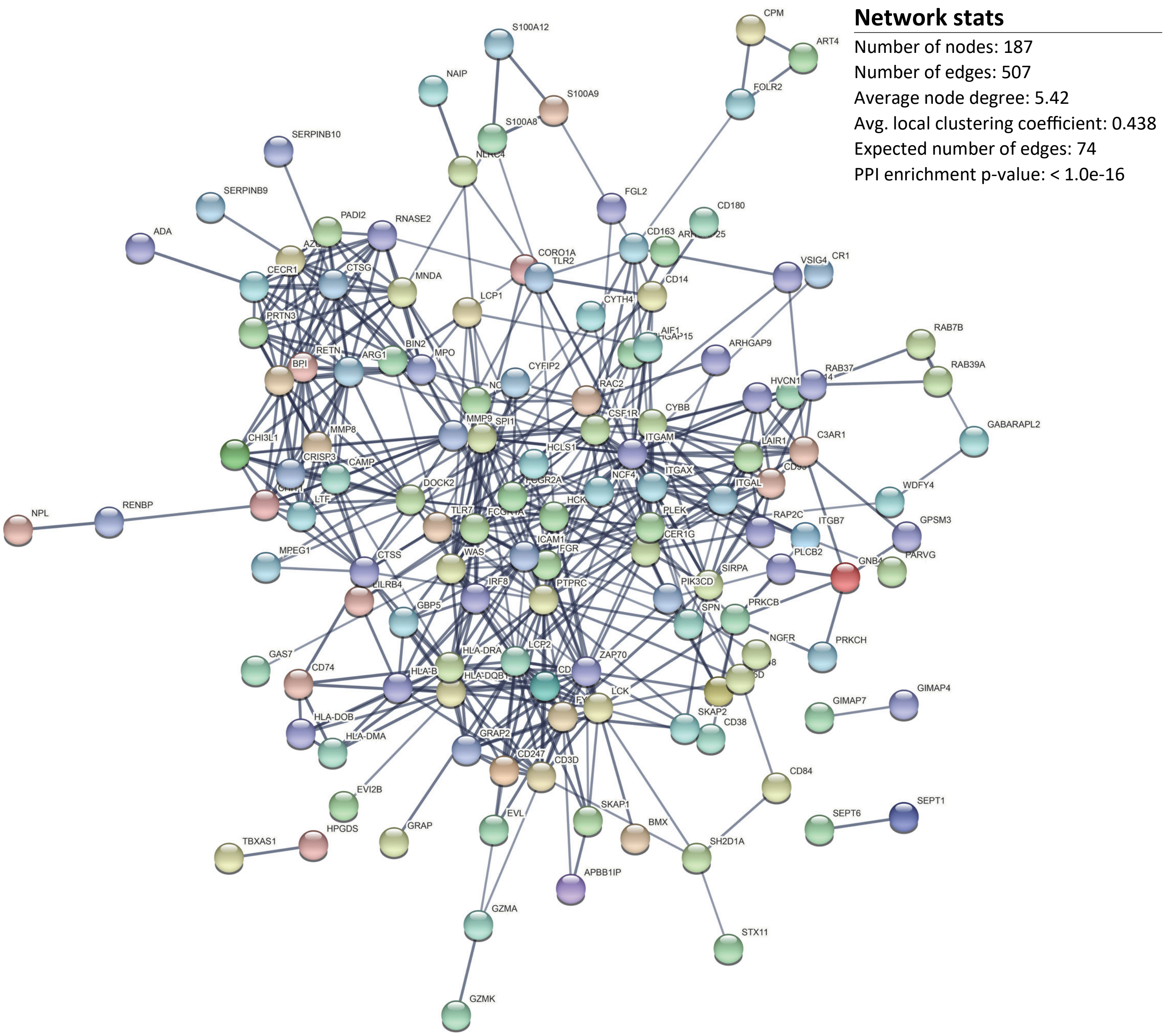

Figure S11. STRING Protein-protein interaction (PPI) network of module M5.The active interaction sources used were Experiments, Databases, co-expression and co-occurrence. The minimum required interaction score was set to a high confidence (0.700) as specified by the string database (https://string-db.org/stringdb.org).

Figure S12

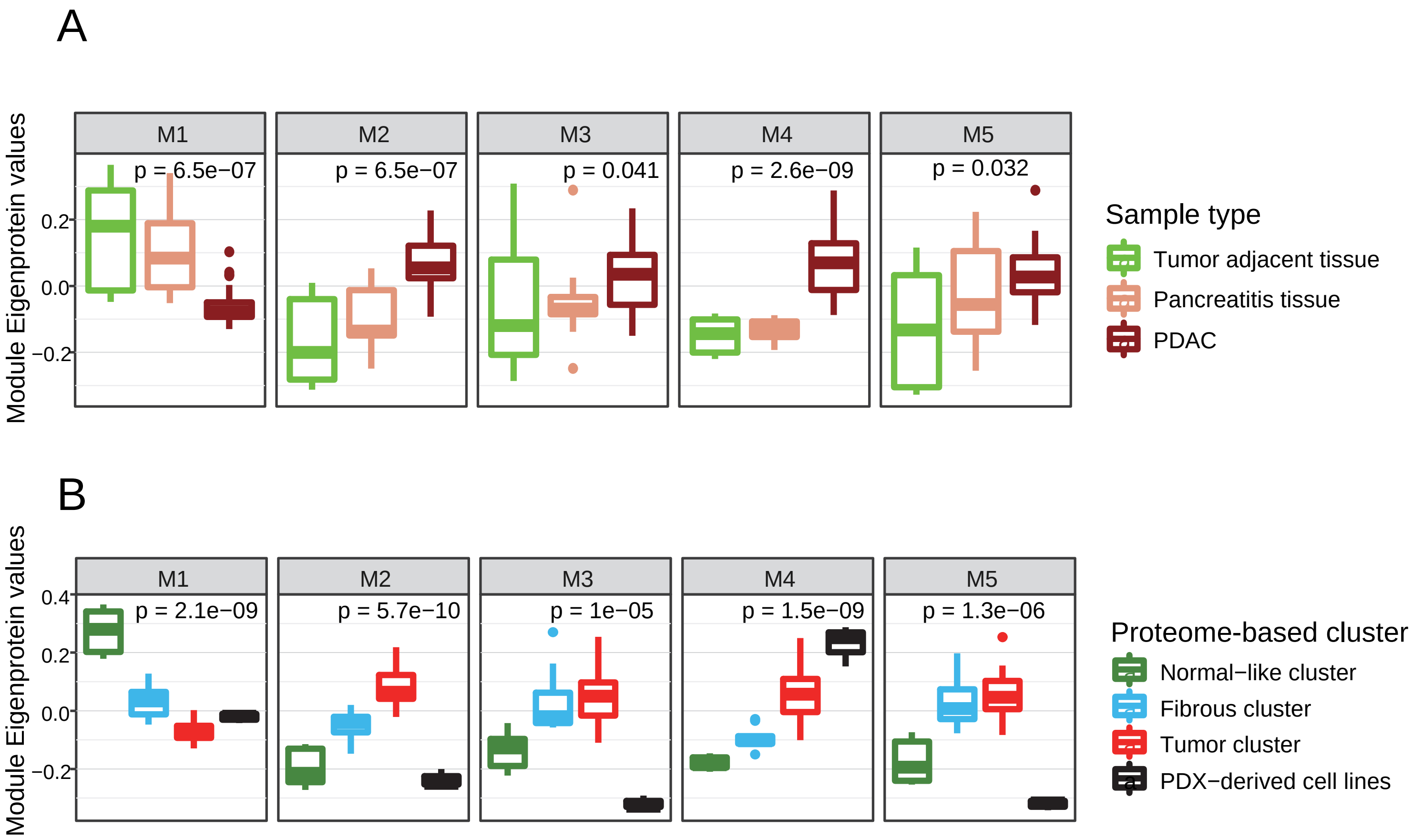

Figure S12. Boxplot illustrating association of the Module Eigenprotein values of each module to A. sample type; B. proteome-based clusters including PDX-derived cell lines. The p-value denotes significance by Kruskal-Wallis test.

Figure S13

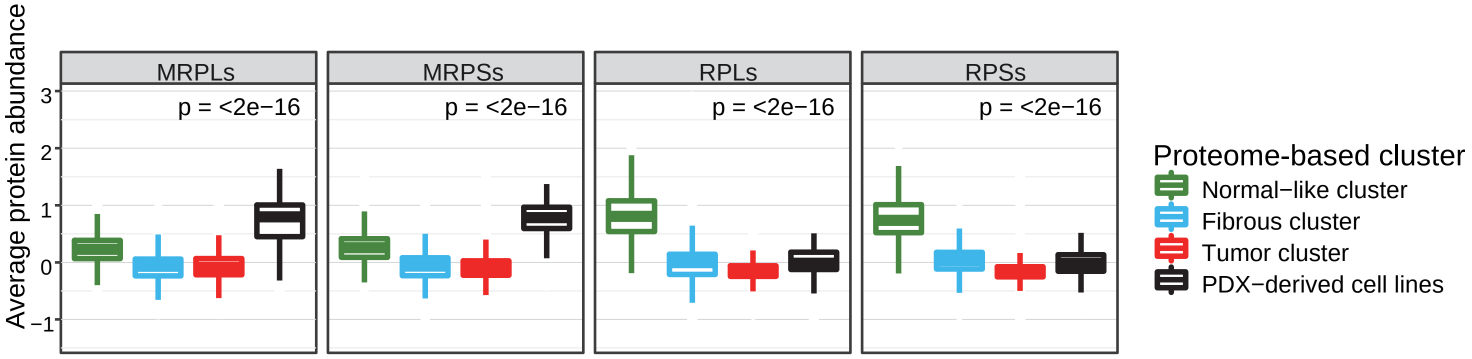

Figure S13. Boxplot comparing average protein abundance of the cytosolic and mitochondrial ribosomal proteins (RPLs, RPSs, MRPLs, MRPSs) in proteome-based clusters including PDX-derived cell lines. The p-value denotes significance by Kruskal-Wallis test.

Figure S14

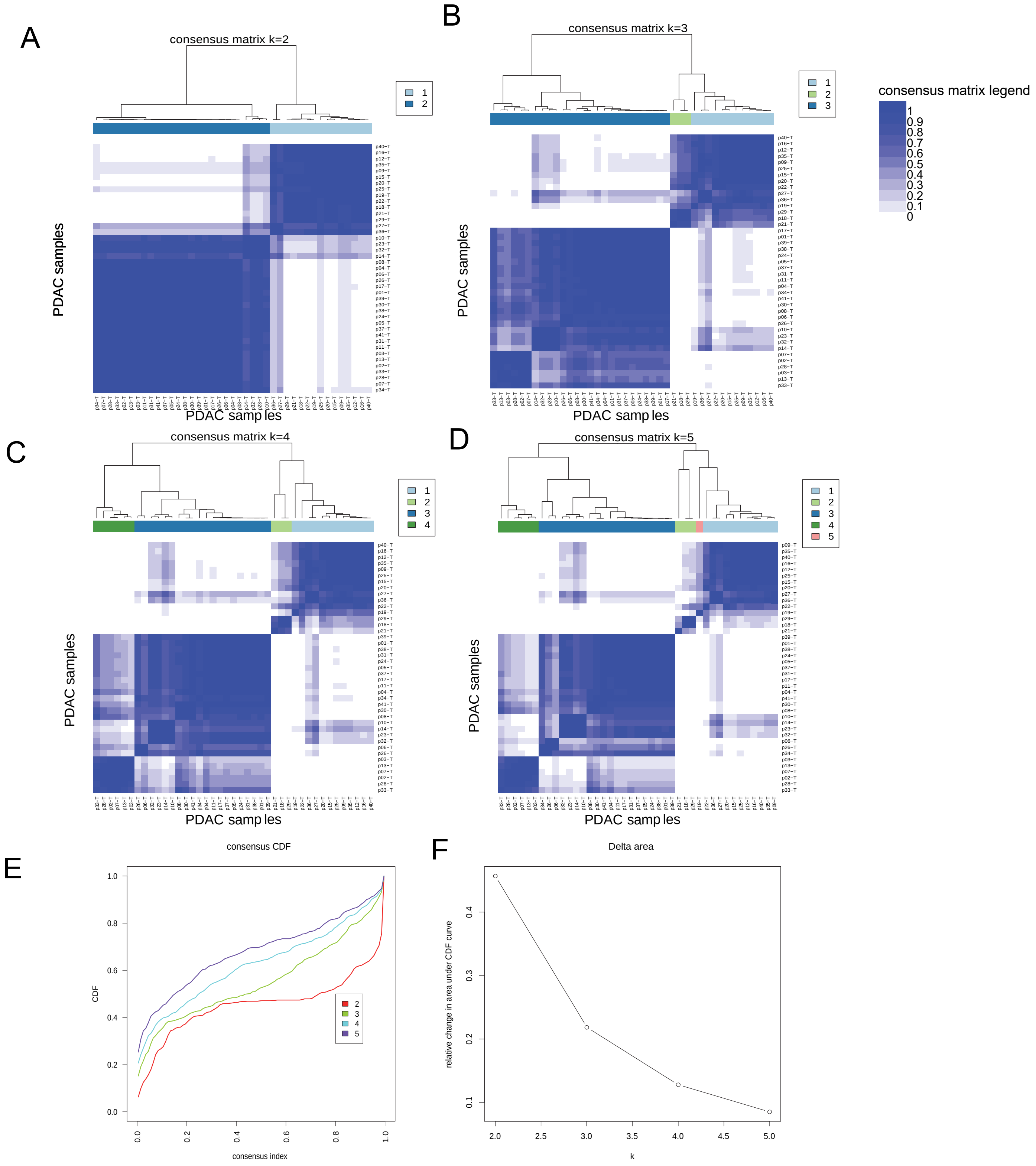

Figure S14. Consensus clustering of PDAC samples using proteins of module M2 using different k values, **A.** k = 2; **B**. k = 3; **C.** k = 4; **D.** k = 5. **E.** Plot showing the cumulative distribution functions (CDF) of the consensus matrix for each k (indicated by colors), estimated by a histogram of 100 bins. **F.** Plot showing the relative change in area under the CDF curve when k increases to k from k−1. For k = 2, there is no k-1, so the total area under the curve rather than the relative increase is plotted.

Figure S15

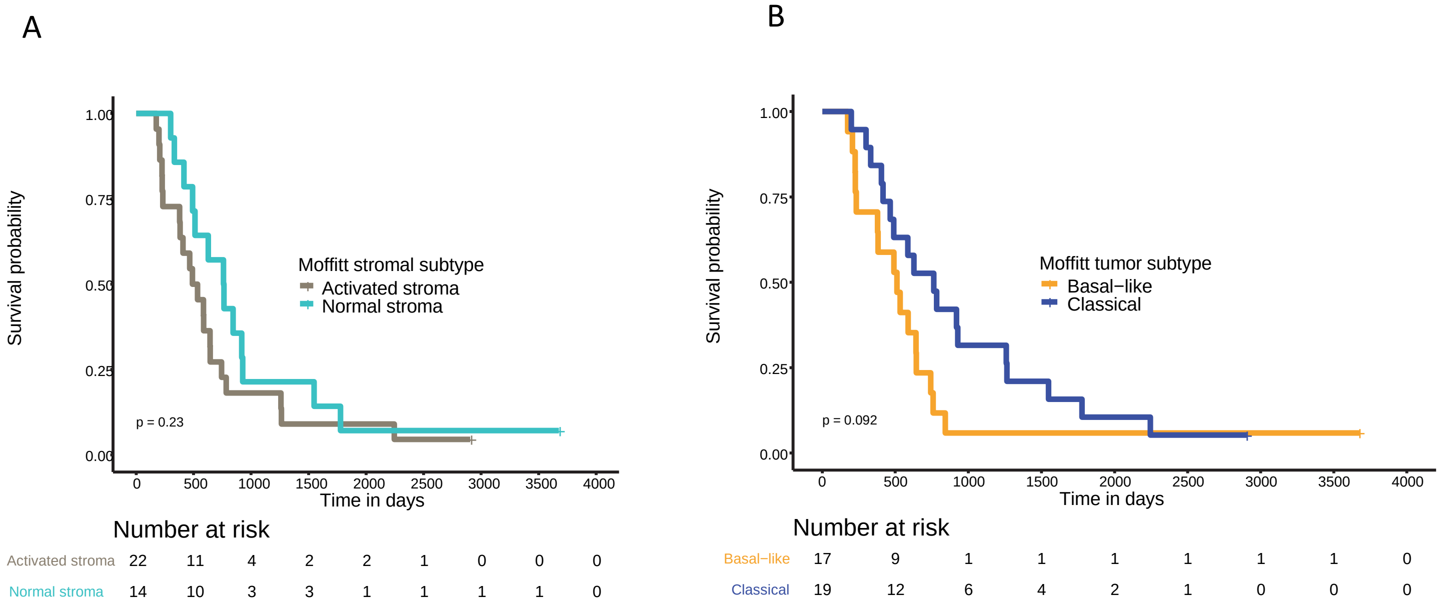

Figure S15. Kaplan-Meier curve showing overall survival trends in the transcriptomics-based A. Moffitt's stromal subtypes; B. Moffitt's tumor subtypes.
